# Genome-wide transcriptome analysis reveals small RNA profiles involved in early stages of stolon-to-tuber transitions in potato under photoperiodic conditions

**DOI:** 10.1186/s12870-018-1501-4

**Published:** 2018-11-16

**Authors:** Kirtikumar Ramesh Kondhare, Nilam Namdeo Malankar, Ravi Suresh Devani, Anjan Kumar Banerjee

**Affiliations:** Biology Division, Indian Institute of Science Education and Research (IISER) Pune, Dr. Homi Bhabha Road, Pune, Maharashtra 411008 India

**Keywords:** Stolon, Potato, MicroRNAs, tasiRNAs, Phased siRNAs, Photoperiod

## Abstract

**Background:**

Small RNAs (sRNAs), especially miRNAs, act as crucial regulators of plant growth and development. Two other sRNA groups, trans-acting short-interfering RNAs (tasiRNAs) or phased siRNAs (phasiRNAs), are also emerging as potential regulators of plant development. Stolon-to-tuber transition in potato is an important developmental phase governed by many environmental, biochemical and hormonal cues. Among different environmental factors, photoperiod has a major influence on tuberization. Several mobile signals, mRNAs, proteins and transcription factors have been widely studied for their role in tuber formation in potato, however, no information is yet available that describes the molecular signals governing the early stages of stolon transitions or cell-fate changes at the stolon tip before it matures to potato. Stolon could be an interesting model for studying below ground organ development and we hypothesize that small RNAs might be involved in regulation of stolon-to-tuber transition process in potato. Also, there is no literature that describes the phased siRNAs in potato development.

**Results:**

We performed sRNA profiling of early stolon stages (4, 7 and 10 d) under long-day (LD; 16 h light, 8 h dark) and short-day (SD; 8 h light, 16 h dark) photoperiodic conditions. Altogether, 7 (out of 324) conserved and 12 (out of 311) novel miRNAs showed differential expression in early stolon stages under SD vs LD photoperiodic conditions. Key target genes (*StGRAS, StTCP2/4* and *StPTB6*) exhibited differential expression in early stolon stages under SD vs LD photoperiodic conditions, indicative of their potential role in tuberization. Out of 830 *TAS-like* loci identified, 24 were cleaved by miRNAs to generate 190 phased siRNAs. Some of them targeted crucial tuberization genes such as *StPTB1, POTH1* and *StCDPKs*. Two conserved *TAS* loci, referred as *StTAS3* and *StTAS5*, which share close conservation with members of the Solanaceae family, were identified in our analysis. One *TAS-like* locus (*StTm2*) was validated for phased siRNA generation and one of its siRNA was predicted to cleave an important tuber marker gene *StGA2ox1*.

**Conclusion:**

Our study suggests that sRNAs and their selective target genes could be associated with the regulation of early stages of stolon-to-tuber transitions in a photoperiod-dependent manner in potato.

**Electronic supplementary material:**

The online version of this article (10.1186/s12870-018-1501-4) contains supplementary material, which is available to authorized users.

## Background

Small RNAs (sRNAs) have emerged as crucial regulators of plant growth and development, as well as in different abiotic and biotic stress responses [1–3]. Based on their biogenesis and functional modes, sRNAs are classified into two major groups: microRNAs (miRNAs) and short-interfering RNAs (siRNAs) [4]. miRNAs are ~ 21 nt long single stranded endogenous sRNAs [5], whereas siRNAs (20–22, 24 nt long) are produced from double stranded RNAs [2] Phased siRNAs (phasiRNAs) or trans-acting siRNAs (tasiRNAs) are special types of siRNAs mainly identified in plants [4] that play crucial roles in meristem initiation, adaxial-abaxial leaf polarity specification, leaf/floral development and patterning, root development [6–9], reproductive development and drought stress [10–12]. *TAS* or *PHAS* loci encode for respective precursors, which are cleaved by specific miRNAs to form a double stranded RNA through RNA-dependent RNA polymerase 6. These double stranded RNAs are further cleaved by Dicer-like 4 to generate 21–22 nt siRNAs that perform gene silencing similar to miRNAs [13–15]. Both tasi and phasiRNAs are generated in phased pattern from their transcripts. PhasiRNAs target the loci from which they are generated and the respective gene family members, whereas tasiRNAs have been demonstrated to function in trans- manner (i.e. targeting unrelated gene transcripts) [2, 12]. Hence, phased siRNAs producing loci cannot be categorized as *TAS* loci until the function of siRNAs generated from it has been proven.

Potato is one of the most important food crops and potato tuber development is of great research interest for understanding the molecular mechanism of tuber yield. Tuberization is a highly complex developmental process that involves interactions between environmental, biochemical, and genetic factors [16, 17]. Under inductive photoperiodic conditions, a stolon passes through several stages of developmental transitions starting from sub-apical hook formation, followed by swelling at the sub-apical region, development of the mini-tuber and tuber maturation [18, 19]. At the onset of tuber formation, it is likely that a dynamic change in regulatory network would govern the plane of cell division from transverse to longitudinal, followed by random cell divisions that result in swelling at sub-apical regions of the stolon [18, 19]. Although several mobile signals, mRNAs, proteins and transcription factors (TFs) have been widely studied for their role in tuber formation in potato [20, 21], no information is yet available that describes the molecular signals governing the early stages of stolon transitions or cell-fate changes at the stolon tip. We speculate that sRNAs could play a major role in this process.

Two earlier studies have reported sRNA profiling of stolons [22, 23]; however, these were limited to identification of miRNAs involved in overall tuber formation and did not emphasize the influence of photoperiod during early stages of stolon development. To identify the novel sRNAs potentially involved in early stages (4, 7 and 10 days) of stolon-to-tuber transitions in a photoperiod-dependent manner, we undertook a deep-sequencing approach using stolon samples of a photoperiod-sensitive potato cultivar (*S. tuberosum* ssp. *andigena*) under long-day (LD) and short-day (SD) photoperiodic conditions. Our analysis revealed 21 conserved/novel miRNAs to be differentially expressed in stolons under LD/SD conditions. Differential expression of select miRNA target genes indicated their potential role in early stages of stolon transitions. Additionally, we identified 24 putative *TAS-like* loci, and 190 phased siRNAs generated from these loci that targeted several tuberization genes (*StPTB1, POTH1, StCDPKs*) [24–27], suggesting their crucial role in stage-specific regulation of stolon development. Overall, these findings advance our understanding of the dynamics of sRNAs and their target genes in controlling early stolon/tuber transition in potato.

## Methods

### Stolon sample collection, RNA isolation and sRNA sequencing

Potato plants (*S. tuberosum* ssp. *andigena* 7540), grown in soil for 8 weeks under long-day (LD; 16 h light, 8 h dark) conditions, were subjected to short-day (SD; 8 h light, 16 h dark) or continued in LD photoperiod at 22 °C in a growth chamber (Percival Scientific). Stolon apexes (1 cm) were harvested from 4, 7 and 10 days post LD/SD induction. We have chosen the early time points (4, 7, and 10 d) of stolon development because the crucial changes in the plane of cell division at the subapical region of the stolon occurs following the onset of tuber inductive signals [18, 19]. Samples were pooled from 4 independent plants off 8 plants forming two biological replicates at each time point. The early stages of stolon development at these three-time points are shown in Additional file 1: Figure S1. For small RNA sequencing, all six samples from LD/SD inductions were processed in duplicates. Altogether, 12 RNA samples were sequenced. Total RNA was extracted with RNAiso Plus (Takara-Clontech). RNA concentration and purity were estimated using the Nanodrop Spectrophotometer and subsequently, RNA integrity was checked using Agilent Bioanalyzer chip. Small RNAs were purified with mirPremier™ microRNA Isolation Kit. sRNA profiles and miRNA contents were also checked on the Bioanalyzer. To identify miRNAs involved in early stages of stolon-to-tuber transitions (4, 7, and 10 d) in potato, 12 sRNA libraries were prepared independently, sequenced on the Illumina platform and the FastQC reports were obtained.

### Processing of raw reads and miRNA identification

Raw read sequences were processed and filtered through several criteria to identify conserved and novel miRNAs using mirPRo, an open-source standalone program (https://sourceforge.net/projects/mirpro/) [28] and mapped onto the potato reference genome (*Solanum tuberosum* v4.03) (https://phytozome.jgi.doe.gov/pz/portal.html) using the Novoalign sequence alignment tool with no mismatches. Novoalign allows perfect mappings to obtain the accurate positions of mature miRNAs in corresponding hairpins for downstream analysis.

### Differential expression analyses and prediction of putative target genes

To identify differentially expressed conserved and novel miRNAs between LD vs SD photoperiod at three early stages of stolon development (LD4 vs SD4, LD7 vs SD7 and LD10 vs SD10), we used R package DESeq2 program (http://bioconductor.org/packages/release/bioc/html/DESeq2.html). For this analysis, count data obtained from mirPRo was provided as an input. MA scatter plot was made for differentially expressed conserved and novel microRNAs for three different LD and SD pairwise comparisons: LD4 vs SD4, LD7 vs SD7 and LD10 vs SD10 comparisons (Additional file 1: Figure S2). Moreover, clustered dendrograms were generated for top 30 representative conserved as well as novel miRNAs from all 12 stolon libraries (Additional file 1: Figure S3). Precursor structures of three novel and three conserved miRNAs were predicted using the mfold Web Server (Additional file 1: Figure S4). FASTA files containing both conserved and novel miRNAs were subjected to psRNA target finder (plantgrn.noble.org/psRNATarget) to predict their putative targets using default parameters with expectancy cut off at E ≤ 3.0 [29].

### Gene ontology

Gene Ontology (GO) annotation was performed using the Blast2GO software v1.3.3 [30, 31] for predicted targets of conserved and novel miRNAs to gain a better understanding of their functions in potato. The FASTA file containing the transcript sequences of all miRNA targets were cloud-blasted using the BlastX program against non-redundant protein database (NCBI) in the Blast2GO software (parameters for cloud-blast: sequence length ≥ 100 bp; number of blast hits, 20; e-value, 10; HSP length cut-off, 33). The mapping tool was used to obtain GO information from retrieved database matches. GO term mapping was done with a sequence length ≥ 100 bp. Annotation of all sequences was performed using the annotation tool against filter GO by taxonomy to green plants, with the following parameters: sequence length ≥ 150 bp; e-value Hit Filter set to 3; annotation cutoff set to 25; GO weight set constantly to 5. GO term-based classification charts were also generated using the Blast2GO software.

### cDNA synthesis and real-time analysis

For validation of miRNAs, total RNA was isolated from the powdered aliquot of the same stolon samples subjected to the deep-sequencing. Four micrograms (4 μg) of total RNA was reverse-transcribed in a 40 μl reaction with miRNA specific stem-loop primers (STPs) using SuperScript-IV reverse transcriptase (SS-IV RT) (Invitrogen) following the previously published protocol [32]. Quantitative RT-PCR (qRT-PCR) for 14 differentially expressed miRNAs (conserved and novel) was performed using miRNA specific forward primer (FP) and universal reverse primer (RP) (Additional file 2: Table S1) in a CFX96 Real-Time System (BIO-RAD). Similarly, for normalization, a non-coding small nuclear RNA *U6* was reverse transcribed by *U6* specific STP primer and was amplified in qRT-PCR reaction by *U6* specific FP and universal RP. All the PCRs were carried out using TAKARA SYBR® green master mix (Takara) and incubated at 95 °C for 30 s, followed by 40 cycles of 95 °C for 5 s, 56 °C for 15 s and 72 °C for 15 s. PCR specificity was checked by melting curve analysis, and data were analysed using the 2^–ΔΔCt^ method [33]. The PCR products were further run on 3% agarose gel to confirm the presence of a single band of expected size. Similarly, for miRNA target validation, 4 μg of total RNA was reverse-transcribed by SS-IV RT in a 40 μl reaction using oligo(dT) primers. qRT-PCR was performed using respective gene specific FP and RP (Additional file 2: Table S1). *EIF3e* was used for normalization of miRNA target genes [34].

### Cleavage site mapping

A modified 5’ RNA Ligase-Mediated Rapid Amplification of Complementary DNA Ends (5′ RLM-RACE) was performed using the First Choice RLM-RACE kit (Ambion) to map the cleavage site on predicted targets for two conserved miRNAs. Two predicted target genes *StGRAS* (target of miR479) and *StGAMYB* (target of miR319b) were selected for this purpose. Total RNA from stolon samples at 4, 7, and 10 d LD/SD time points was mixed in equal concentration and ligated to RNA adapter without any enzymatic pre-treatments. cDNA synthesis was performed by SS-IV RT using corresponding gene-specific outer RPs. Primary PCR was conducted with adapter-specific outer FP and gene-specific outer RP (RPo). Similarly, secondary PCR was conducted with adapter-specific inner FP and gene-specific inner RP (RPi) (Additional file 2: Table S1). The PCR products were then cloned into a sub-cloning vector pGEM-T Easy (Promega) and were sequenced to identify the miRNA cleavage sites. *StARF1*0, which is a target of miR160 in potato, was used as a positive control in 5′ RLM-RACE assay [35].

### Prediction of phased siRNAs and their putative targets

To identify *TAS-like* loci and phased siRNAs in the potato genome, sRNA sequences from all 12 stolon libraries were mapped to potato reference genome (*Solanum tuberosum* v4.03) using TA-SI prediction tool from UEA small RNA workbench (version 3.2; http://srna-workbench.cmp.uea.ac.uk/) with default parameters at *p* < 0.0001 [36]. This tool is an implementation of an algorithm from Chen et al. [37]. In addition, to identify binding sites of conserved and novel miRNAs on these *TAS-like* loci, these sequences were given as an input to the psRNA target finder, and potential phased siRNAs producing loci were identified. The genomic locations of *TAS-like* loci were retrieved from the PGSC database, which helped us to denote their presence in the exonic or intergenic regions on the chromosomes.

Phased siRNAs predicted from 24 *TAS-like* loci were subjected for psRNA target finder (E < 3.0) to identify putative target genes of these siRNAs. GO analysis was performed for functional annotation of siRNA target genes as described above. GO terms were further subjected to enzyme code mapping and KEGG pathways were obtained.

Two putative *TAS-like* loci present within the exonic (genic) regions were chosen for their detection. RT-PCR analysis was performed from cDNA synthesised from stolon samples (4 μg of total RNA) using primers on either side of the predicted cleavage sites in their transcript sequences, respectively (Additional file 2: Table S1).

The expression profiles of *StTm2* and *StPHO2* were checked at 4, 7 and 10 d time points under LD and SD photoperiodic conditions. *EIF3e* was used for normalization of real-time data. Similarly, RT-PCR analysis was conducted for detection of phased siRNAs generated from these loci (two from each locus) using STP specific cDNA as template, and siRNA specific FP and universal RP (Additional file 2: Table S1). Amplified siRNA sequences were cloned into a sub-cloning vector pGEM-T Easy (Promega) and sequence verified. qRT-PCR analysis was conducted for four siRNAs generated from these two loci at 4, 7 and 10 d time points under LD and SD photoperiodic conditions. *U6* was used for normalization of siRNA real-time data. To map the cleavage sites in *StTm2* transcript (predicted to be cleaved by miR6026-3p), a modified 5′ RLM-RACE was performed using the First Choice RLM-RACE kit (Ambion) as described above.

In order to identify the presence of conserved *TAS* loci in potato, *TAS* sequences from *Arabidopsis thaliana*, *Nicotiana tabacum*, and *Solanum lycopersicum* were aligned to all the *TAS* loci predicted in our analysis using BLAST. Global alignment of *TAS* loci identified from potato was carried out using MUSCLE [38]*.* The alignment file was further processed using BoxShade server (version 3.21, written by Hofmann and Baron).

## Results

### Analysis of sRNA population

**Fig. 1:**
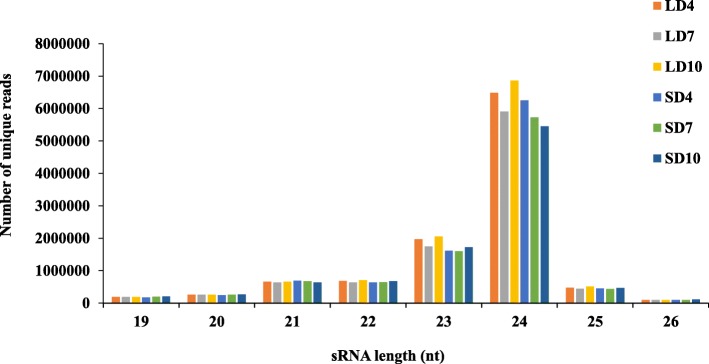
Size distribution of unique small RNA sequences identified in all twelve small RNA libraries. Samples LD4, LD7 and LD10 represents 4, 7 and 10 days during long-day induction respectively, whereas samples SD4, SD7 and SD10 represents 4, 7 and 10 days during short-day induction respectively

Altogether 188 million final clean reads were obtained out of 220 million raw reads after quality filtering and adapter trimming. Out of the final clean reads, 84.43% mapped to the genome. Approximately, 12.68% (23,848,593) and 0.94% (1,759,128) were mapped to the known and novel hairpin sequences, respectively (Table 1). The size distribution analysis of these small RNA sequences showed that majority of the reads were 21 to 25 nt in length (Fig. 1). The 24 nt long sequence size class was the most abundant in all the libraries, followed by 23, 21, 22 and 25 nt classes (Fig. 1).

**Table 1:** Summary of reads from twelve stolon small RNA libraries after adapter removal and filtering. The individual read-based statistics for whole miRNA analysis is also represented

Types of reads/miRNAs	No. of reads/miRNAs
Total number of sequences (raw reads)	220,646,836
Final clean reads after adapter trimming	188,071,173
Percentage of final clean reads mapped to the genome	84.43%
Final clean reads mapped to conserved pre-miRNAs (hairpins)	23,848,593
Final clean reads counted as conserved mature miRNAs	8,690,556
Conserved mature miRNAs in all samples	324
Conserved miRNA families in all samples	114
Final clean reads mapped to novel pre-miRNAs (hairpins)	1,759,128
Final clean reads counted as novel mature miRNAs	1,442,393
Novel mature miRNAs in all samples	311

### Identification of conserved and novel miRNAs

Deep-sequencing analyses using miRPRo showed that among total raw reads, on an average 4.6% of final clean reads were counted as conserved mature miRNAs, whereas 0.76% of final clean reads were counted as novel mature miRNAs. Overall, our study from 12 stolon libraries detected 324 conserved miRNAs belonging to 114 miRNA families, and 311 novel miRNAs (Table 1; Additional file 3: Table S2). Out of 311 novel miRNAs that were identified, star strand was detected for 270 miRNAs. Further, 173 miRNAs had star strand detected in at least two sequencing samples (Additional file 3: Table S2; Novel miRNAs sheet).

### Differential expression analyses of miRNAs

Among the conserved miRNAs, only 7 were found to be differentially expressed between LD4 and SD4 conditions (LD4 vs SD4), whereas two miRNAs that were found to be differentially expressed in LD4 vs SD4 conditions, were also differentially expressed between LD10 and SD10 conditions (LD10 vs SD10) (Table 2; Additional file 4: Table S3; Additional file 1: Figure S2). For novel miRNAs, 10 were found to be differentially expressed between LD4 and SD4 comparisons (LD4 vs SD4), whereas two were differentially expressed between LD10 and SD10 conditions (LD10 vs SD10) (Table 2; Additional file 5: Table S4; Additional file 1: Figure S2). No miRNAs showed differential expression for LD7 and SD7 comparisons (LD7 vs SD7) among both conserved and novel miRNAs (Table 2; Additional file 1: Figure S2). Thus, in total, 19 miRNAs (7 conserved miRNAs and 12 novel miRNAs) were differentially expressed at either LD4 vs SD4 or LD10 vs SD10 comparisons.

**Table 2:** List of differentially expressed conserved as well as novel miRNAs identified from LD4 vs SD4 and LD10 vs SD10 stolon transition time points comparisons. Number of putative targets predicted by psRNA target for each miRNA is also represented with (E < 3.0). None of the miRNAs were differentially expressed at LD7 vs SD7 time point comparison

	miRNA	log2FoldChange	*p* value	p adjusted value	No. of predicted targets (E < 3.0)
Conserved miRNAs					
LD4 vs SD4	stu-miR399g-3p	−1.45	0.0006	0.0196	0
	stu-miR482d-3p	−0.71	0.0003	0.0101	18
	stu-miR319-3p	0.69	0.0001	0.0070	11
	stu-miR8006-5p	0.80	0.0001	0.0070	3
	stu-miR479	1.21	0.0000	0.0010	3
	stu-miR477b-5p	1.54	0.0008	0.0210	4
	stu-miR477a-5p	1.73	0.0000	0.0001	4
LD10 vs SD10	stu-miR477b-5p	1.79	0.0003	0.0526	4
	stu-miR477a-5p	2.38	0.0000	0.0001	4
Novel miRNAs					
LD4 vs SD4	stu-novel-miR302	−1.39	0.0000	0.0000	6
	stu-novel-miR93	−0.65	0.0002	0.0036	3
	stu-novel-miR94	−0.65	0.0002	0.0036	3
	stu-novel-miR221	−0.62	0.0055	0.0353	3
	stu-novel-miR278	0.53	0.0041	0.0288	0
	stu-novel-miR279	0.53	0.0041	0.0288	0
	stu-novel-miR276	0.61	0.0022	0.0202	17
	stu-novel-miR277	0.61	0.0022	0.0202	17
	stu-novel-miR40	0.66	0.0003	0.0036	0
	stu-novel-miR147	1.01	0.0000	0.0000	3
LD10 vs SD10	stu-novel-miR139	1.34	0.0002	0.0289	0
	stu-novel-miR206	1.34	0.0001	0.0289	4

### Time-course expression analysis of miRNAs

**Fig. 2:**
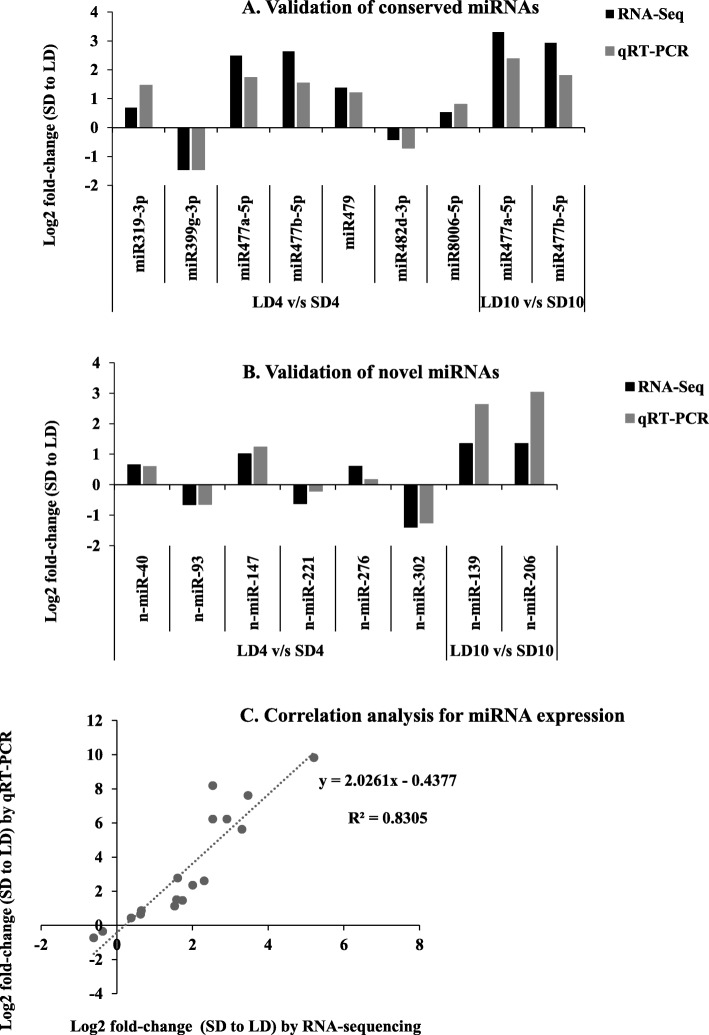
Validation of deep-sequencing data by qRT-PCR analysis. Differentially expressed selective miRNAs were chosen for validation. **a** Validation of conserved miRNAs. **b** Validation of novel miRNAs. *U6* was used as a reference gene for normalization of qRT-PCR data. The expression level of miRNAs under LD at corresponding time point was considered as 1 for measurement of relative expression at respective SD time-points. Data is represented as means from two biological replicates and three technical replicates. **c** A correlation analysis shows the relationship of miRNAs expression between the RNA-seq and qRT-PCR analysis

In total, 15 miRNAs (7 conserved and 8 novel), that were differentially expressed at either LD4 vs SD4 or LD10 vs SD10 comparisons, were tested by qRT-PCR for their validation. The expression data for all 15 miRNAs were highly correlated (*R*^2^ = 0.8305) between the RNA-seq and qRT-PCR analysis (Fig. 2), suggesting the reliability of the deep-sequencing data.

**Fig. 3:**
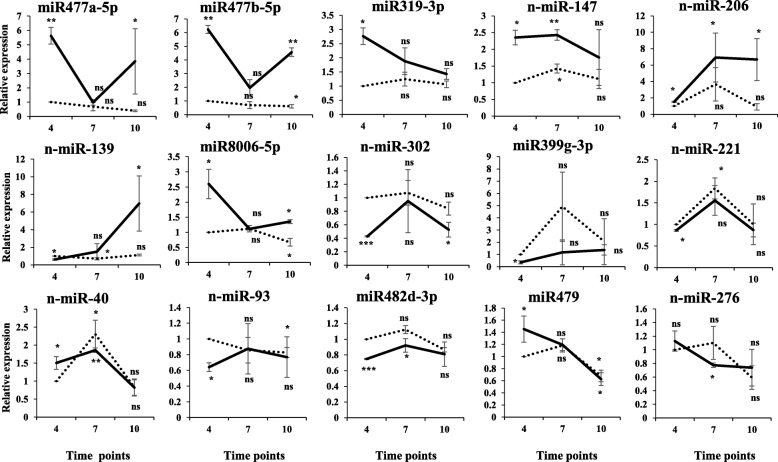
Expression analysis of novel and conserved miRNAs at 4, 7 and 10 d time-points under LD vs SD photoperiod conditions. For qRT-PCR analysis, *U6* was used as a reference gene for normalization and the expression level of miRNAs at LD4 was considered as 1 for measurement of relative expression at other time-points. Error bars represent standard error of means from two biological replicates. For graphs, a dotted line represents LD condition, whereas a thick line represents SD condition. Asterisks (one and two) indicate significant differences at *p* < 0.05 and *p* < 0.01, respectively, using a Student’s t-test. ns = not significant at *p* < 0.05

Two conserved miRNAs (miR477a-5p and miR477b-5p) showed significantly higher expression levels at SD4 and SD10 d time-points compared to the LD4 time-point, whereas miR477b-5p exhibited significant reduction in its expression at LD10 compared to LD4 (Fig. 3). MiR319-3p expression was significantly higher at SD4 compared to LD4, however its expression remained unchanged at other time points tested (Fig. 3). One novel miRNA (n-miR-147) showed a significant increase in its expression level under SD conditions compared to LD conditions at 4 and 7 d time-points (Fig. 3). The expression of a novel miRNA (n-miR-206) was significantly high under SD conditions than LD conditions at all three time points tested, but the difference was more distinct at 7 and 10 d than the 4 d time-point (Fig. 3).

Novel miRNA, n-miR-139, exhibited significantly low expression at SD4 compared to LD4, but its expression significantly increased towards SD10 d time point (Fig. 3). MiRNA (miR8006-5p) showed a significant increase in its expression under SD conditions compared to LD conditions at 4 and 10 d time point compared to LD4; however, the expression level remained unchanged at 7 d time-point (Fig. 3). A conserved miRNA (miR482d-3p) and two novel miRNAs (n-miR-302 and n-miR-93) exhibited a distinct reduction in their expression levels at 4 d time-point under SD compared to LD conditions (Fig. 3). Moreover, miR482d-3p and n-miR-302 also showed reduced expression at SD7 and SD10 d time points, respectively (Fig. 3). Although miR479 showed a significant increase in its expression level under SD conditions at 4 d time-point, its relative expression levels remained significantly low under both LD and SD conditions at 10 d time-point (Fig. 3). A novel miRNA, n-miR276, exhibited a reduction in its expression level at 7 d time-point under SD conditions compared to LD4 (Fig. 3). Another novel miRNA (n-miR-40) had significantly high expression at SD4 as well as LD7 and SD7 time points when compared with LD4 (Fig. 3). In case of miR399g-3p, a significant reduction in its expression was observed under SD4 conditions than LD4 conditions (Fig. 3). A novel miRNA, n-miR221, showed a significant reduction in its expression at 4 d time-point under SD conditions; however, the expression level was significantly high at LD7 compared to LD4 time-point (Fig. 3).

### GO analysis for predicted targets of miRNAs

**Fig. 4:**
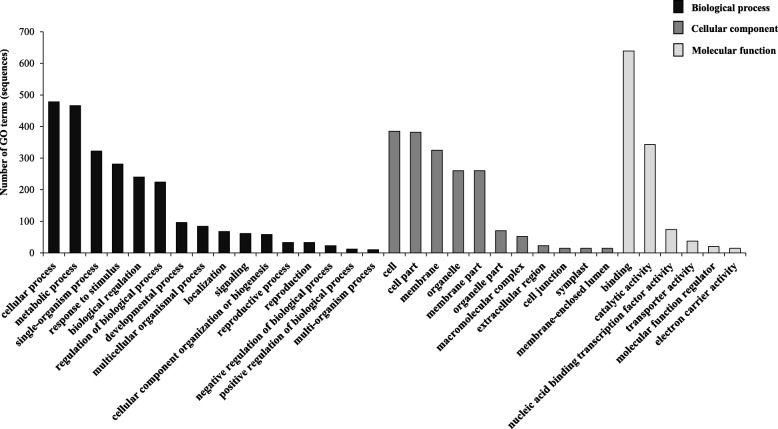
Gene Ontology (GO) categorization for predicted targets of novel and conserved miRNAs identified. GO terms were categorized into biological process, cellular components and molecular functions. GO terms with > 10 sequences were considered for preparing a graph in each category

Altogether, 1414 putative targets were predicted for 653 conserved/novel miRNAs (Additional file 6: Table S5). For differentially expressed conserved and novel miRNAs, several interesting genes were identified as putative targets, such as TEOSINTE BRANCHED 1, cycloidea and PCF transcription factors TCP2 and TCP4 (both targeted by miR319-3p), GRAS family transcription factors DELLA and SCARECROW (targeted by miR477a/b-5p and miR479, respectively), protein phosphatase 2c (targeted by miR8006-5p), cytochrome P450 (targeted by novel-miR-147) and methyl-transferase ASHR3 (targeted by novel-miR-221) (Additional file 6 Table S5; Targets of DE miRNAs sheet). GO analysis for 1414 putative targets categorised them into a total of 1970 GO terms. Of which, 1235 GO terms belongs to biological processes, 236 GO terms were included under cellular components, whereas 499 GO terms were categorised to molecular functions (Additional file 7: Table S6; Individual GO types sheet). In the biological process category, cellular, metabolic, response to stimulus, biological regulation, development, localization and signalling were most enriched, whereas several functions such as nucleic-acid binding TF activity, catalytic activity, regulation and electron carrier activity were greatly enriched in molecular functions category (Fig. 4). Moreover, in cellular component category, cell and cell part, membrane and organelle categories were highly enriched (Fig. 4).

Our GO analysis of putative miRNA targets clearly demonstrated that auxin, cytokinin (CK) and gibberellin (GA) metabolism related genes were identified as putative targets of conserved and novel miRNAs (Additional file 7: Table S6; Target gene annotation sheet). For example, auxin biosynthesis gene (indole-3-pyruvate monooxygenase YUCCA8), auxin signaling related genes (TRANSPORT INHIBITOR RESPONSE 1-like), auxin-responsive factors (SAUR71-like, SAUR32-like, ARF10/16) and auxin inducible gene (ARGOS) were identified as miRNA targets (Additional file 7: Table S6; Target gene annotation sheet; yellow highlighted). From CK biosynthesis genes, we identified cytokinin dehydrogenase, LOG10, zeatin O-glucosyltransferase, UDP-glycosyltransferase 708c1 as miRNA targets (Additional file 7: Table S6; Target gene annotation sheet; green highlighted). Additionally, potential CK transporters, such as equilibrative nucleotide transporter 3-like (ENT) and purine permease 1-like (PUP); one CK response regulator (ARR1-like) were also identified (Additional file 7: Table S6; Target gene annotation sheet; green highlighted). GA precursor (GGPP) biosynthesis enzyme such as farnesyl pyrophosphate synthase, and GA signaling regulator (DELLA RGL1-like) (Additional file 7: Table S6; Target gene annotation sheet; blue highlighted) were also identified as miRNA targets. miRNA targets were also from ethylene and abscisic acid (ABA) biosynthesis pathway (Additional file 7: Table S6; Target gene annotation sheet; brown highlighted). Several ABA response related genes, i.e. E3 ubiquitin- ligase LOG2, phosphatase 2C 55, EARLY RESPONSIVE TO DEHYDRATION 15-like and DEHYDRATION-INDUCED 19 homolog 5-like isoform X2, were also identified as targets (Additional file 7: Table S6; Target gene annotation sheet; brown highlighted). Epigenetic modifiers class (methyl- and acetyl-transferases) was also enriched as miRNA targets (Additional file 7: Table S6; Target gene annotation sheet; grey highlighted). Light-mediated response related genes were also found to be the targets of miRNAs e.g. cryptochrome 1, cryptochrome-1-like isoform X1, ultraviolet-B receptor UVR8, transcription factor PHYTOCHROME INTERACTING FACTOR 1 (PIF1-like), B-box zinc finger 19-like (Additional file 7: Table S6; Target gene annotation sheet; red highlighted). Number of genes associated to flowering, growth regulation, Cytochrome P450 like, Argonaut proteins, variety of transcription factors and kinase/phosphatases were also identified as miRNA targets (Additional file 7: Table S6; Target gene annotation sheet).

### MiRNAs and their putative targets relationship

**Fig. 5:**
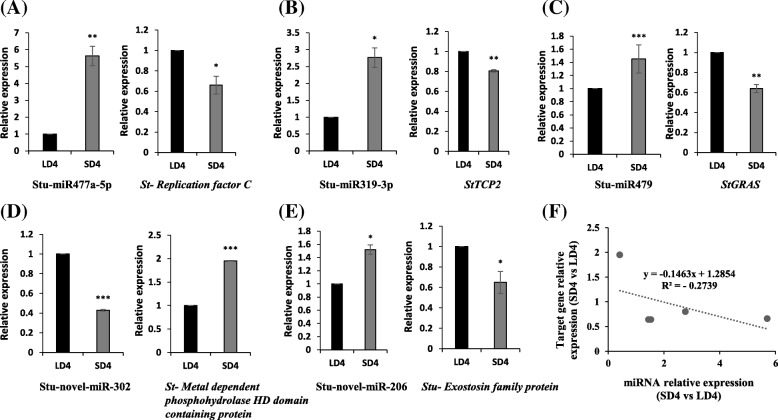
Relationship between miRNAs and their predicted targets by qRT-PCR at 4 d time-point under LD vs SD photoperiod conditions. Conserved miRNAs and their targets - (**a**) Stu-miR477a-5p and its target *replication factor C*, (**b**) Stu-miR319-3p and its target *StTCP2*, (**c**) Stu-miR479 and its target *StGRAS*. Novel miRNAs and their targets – (**d**) Stu-novel-miR302 and its target *metal dependent phophohydrolase HD domain containing protein*, and (**e**) Stu-novel-miR206 and its target *exostosin family protein*. **f** A correlation analysis shows the inverse relationship between miRNA expression and their putative target genes. *U6* and *EIF3e* were used for normalization of miRNAs and target genes, respectively. The expression level of respective miRNA or target gene at LD4 time point was considered as 1 to measure relative expression at SD4 time point. Error bars represent standard error of means from two biological replicates. Asterisks (one, two and three) indicate significant differences at *p* < 0.05, *p* < 0.01, *p* < 0.001, respectively, using a Student’s t-test. ns = not significant at *p* < 0.05

To investigate the correlation between the expressions of conserved or novel miRNAs with their putative targets, the expression levels of five miRNAs (chosen from differential expression analysis; Table 2), and their corresponding target genes were studied by real-time analysis at 4 d time-point under SD and LD conditions (Fig. 5). All five miRNAs showed an inverse correlation (*R*^2^ = − 0.2739) with their respective putative target mRNA levels (Fig. 5f), suggesting the reliability of the miRNA target prediction software used in the analysis. MiRNAs, such as miR477a-5p, miR319-3p, miR479 and n-miR-206 were up-regulated under SD conditions compared to LD photoperiod, whereas their respective targets (*replication factor C* [miR377a-5p], *StTCP*2 [miR319-3p], *StGRAS* [miR479], exostosin family protein [n-miR-206]) were downregulated (Fig. 5a-c; e). In case of n-miR-302, the expression was reduced, whereas its predicted target (*metal dependent phopsphohydrolase HD domain containing protein*) was up-regulated under SD conditions compared to LD photoperiod (Fig. 5d).

### Cleavage site mapping for targets of conserved miRNAs

**Fig. 6:**
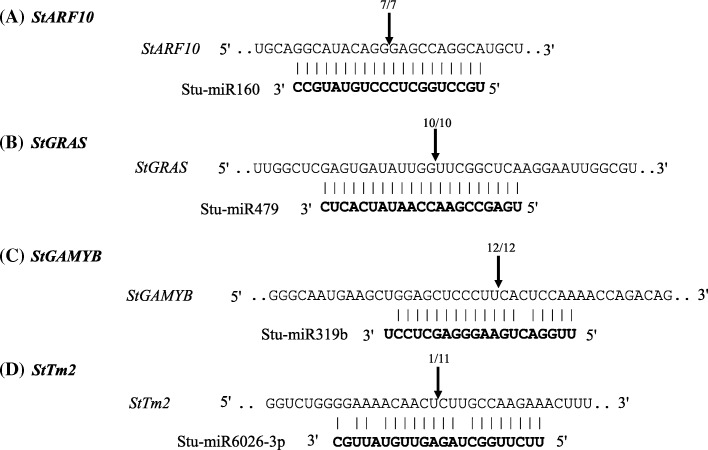
5′ RLM-RACE for cleavage site mapping. Arrows show frequency of 5’ RACE clones showing cleavage sites and numbers represent fractions with proportions of clones showing these cleavage sites. **a** *StARF10*, which is a target of Stu-miR160 was used a positive control [35]. Two conserved miRNAs (stu-miR479 and stu-miR319b) were chosen to map cleavage site on *StGRAS* (**b**) and *StGAMYB* (**c**) transcription factors, respectively. **d** Cleavage site mapping for miR6026-3p is shown on *StTm2 TAS-like* locus. Alignment between mature miRNA (bold) and target gene sequences is shown. The transcript accession IDs are- *StARF10:* PGSC0003DMT400020874; *StGRAS*: PGSC0003DMT400031475; *StGAMYB*: PGSC0003DMT400058426, and *StTm2:* PGSC0003DMT400051269

A modified 5′ RLM-RACE analysis validated *StARF10* as a target of miR160 with high cleavage frequency (7 of 7) at 10th/11th nucleotides position (Fig. 6a). Similarly, *StGRAS* (10 of 10) and *StGAMYB* (12 of 12) were found to be true targets of miR479 and miR319b, respectively (Fig. 6b-c). Moreover, it was found that *StTm2* transcript was cleaved by miR6026-3p with a very low frequency (1/11) (Fig. 6d). Sequencing results for RACE cloning are shown in Additional file 1: Figure S6.

### MiRNA target gene expression analysis

**Fig. 7:**
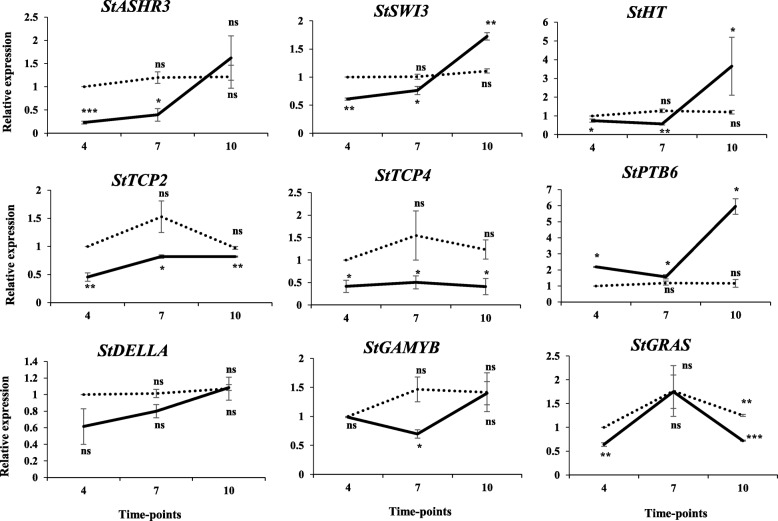
Expression analysis for selected target genes of novel and conserved miRNAs at 4, 7 and 10 d time-points under LD vs SD photoperiod conditions. *EIF3e* was used as a reference gene for normalization. The expression level of each target gene at LD4 was considered as 1 for measurement of relative expression at other time-points. Error bars represent standard error of means from two biological replicates. For graphs, a dotted line represents LD condition, whereas a thick line represents SD condition. Asterisks (one, two and three) indicating significant differences at *p* < 0.05, *p* < 0.01, *p* < 0.001, respectively, using a Student’s t-test. ns = not significant at *p* < 0.05

The expression profiling of nine interesting target genes was studied at 4, 7 and 10 d under LD/SD photoperiodic conditions. Some of these genes were selected based on the available literature about tuberization pathway and their potential involvement in this process. *StASHR3*, *StSWI3* and *StHT* showed a significant reduction in their transcript level under SD at both 4 and 7 d time point, whereas their transcript levels (except *StASHR3*) were significantly high at SD10 time point compared to LD4 (Fig. 7). The mRNA levels of TCP transcription factors (*StTCP2* and *StTCP4*) were significantly reduced at all three points under SD conditions compared to LD4 (Fig. 7). Another miRNA target, *StPTB6* exhibited a significant increase in its expression under SD at all three time points tested, however, the effect was more enhanced at SD10 time point. The transcript abundance of a GA signaling components (*StDELLA* and *StGAMYB*) remained unchanged at all time points tested compared to LD4, except SD7 time point for *StGAMYB*; where its expression was significantly low at SD7. The mRNA level of *StGRAS* was significantly low at SD4 and SD10 time points, whereas it was significantly high under LD10 time point when compared to LD4 (Fig. 7).

### Identification of potential *TAS-like* loci

Our data analysis (at *p* < 0.0001) resulted in identification of 830 putative *TAS-like* loci from all 12 stolon sample libraries. Out of these loci, 275 were found to be present in genic (Additional file 8: Table S7; line 2–276), whereas 555 in intergenic (Additional file 8: Table S7; line 276–831) regions of the potato genome. We observed that many putative *TAS-like* loci reside in genes associated with plant growth and development, such as *PHO2, cytochrome P450 like TBP, auxin related genes (auxin:hydrogen symporter, AUX/IAA 2, TIR1 receptor), serine/threonine protein kinase pk23, P450 mono-oxygenase, F-box family proteins, AGO1–1, squamosa promoter binding protein, WD-repeat protein* and *COP1 homolog* (Additional file 8: Table S7; yellow highlighted). Off 830 *TAS-like* loci, 24 were found to be targeted by either conserved (11) or novel (13) miRNAs, respectively (Table 3). From which, 16 were present in the intergenic regions, whereas 8 loci were present in the genic regions.

**Table 3:** List of *TAS-like* loci predicted to be cleaved by conserved or novel miRNAs. Twenty-four *TAS-like* loci with their genomic locations and miRNA cleaving them are listed. PGSC transcript IDs and annotation for *TAS-like* loci that are present in genic regions is also mentioned. Two *TAS-like* loci present within *StTm2* and *StPHO2* genes (captured in bold) were used for further experimental validations

Sr. No.	*TAS-like* Locus	Genomic location of locus	Locus targeted by miRNA	Gene id	Gene annotation
1	Chr11:458098..458349	Intergenic region	stu-miR5303g	PGSC0003DMT400034561	*Inositol monophosphatase 3*
2	Chr00:19041735..19041986	Intergenic region	stu-miR8005a/b/c	None	None
3	Chr12:27420198..27420449	Intergenic region	stu-miR8008a, stu-miR8009	None	None
4	Chr06:5520530..5520781	Exonic region	stu-miR482c	PGSC0003DMT400022440	*NBS-coding resistance gene protein*
**5**	**Chr02:33977738..33977989**	**Exonic region**	**stu-miR399a-f**	**PGSC0003DMT400076435**	***PHO2***
6	Chr00:14668823..14669074	Intergenic region	stu-miR8005a/c; stu-miR8005b-3p	None	None
7	Chr00:39466733..39466984	Intergenic region	stu-miR8005a/c; stu-miR8005b-3p	None	None
8	Chr00:41890891..41891142	Intergenic region	stu-miR8005a/c; stu-miR8005b-3p	None	None
**9**	**Chr09:15393324..15393575**	**Exonic region**	**stu-miR6026-3p**	**PGSC0003DMT400051269**	***Tm2***
10	Chr02:22364494..22364745	Intergenic region	stu-miR7983-5p	None	*Hydroxyproline-rich glycoprotein*
11	Chr01:88411142..88411393	Intergenic region	stu-miR5303a-f	None	Hypothetical gene of unknown function
12	Chr04:4747066..4747317	Intergenic region	stu-novel-miR-124	None	None
13	Chr09:46314301..46314552	Exonic region	stu-novel-miR-213	PGSC0003DMT400022135	*Disease resistance protein RGA4*
14	Chr10:45893671..45893922	Exonic region	stu-novel-miR-263	PGSC0003DMT400068569	*Cc-nbs-lrr resistant protein*
15	Chr11:42040081..42040332	Intergenic region	stu-novel-miR-272	None	None
16	Chr11:38087726..38087977	Exonic region	stu-novel-miR-272	PGSC0003DMT400018940	*Disease resistance protein R3a*
17	Chr11:6031307..6031558	Intergenic region	stu-novel-miR-276	None	None
18	Chr12:29516380..29516631	Exonic region	stu-novel-miR-52, stu-novel-miR-53, stu-novel-miR-72	PGSC0003DMT400086126	Conserved gene of unknown function
19	Chr02:18433855..18434106	Intergenic region	stu-novel-miR-71	None	None
20	Chr08:54903243..54903494	Intergenic region	stu-novel-miR-171	None	None
21	Chr04:5836408..5836659	Intergenic region	stu-novel-miR-124	None	None
22	Chr12:27420198..27420449	Intergenic region	stu-novel-miR-13	None	None
23	Chr03:44097532..44097783	Exonic region	stu-novel-miR-204	PGSC0003DMT400063143	*Formin 20*
24	Chr08:9967120..9967371	Intergenic region	stu-novel-miR-41, stu-novel-miR-230	None	None

### Identification of phased siRNAs and their target analysis

We identified 59 phased siRNAs generated from 11 *TAS-like* loci triggered by conserved miRNAs (i.e. miR399a-f, miR482c, miR5303a-g, miR6026-3p, miR7983-5p, miR8005a-c, miR8008a, miR8009), whereas 131 phased siRNAs from 13 *TAS-like* loci targeted by novel miRNAs (i.e. novel-miR-13, novel-miR-41, novel-miR-52, novel-miR-53, novel-miR-71, novel-miR-72, novel-miR-124, novel-miR-171, novel-miR-204, novel-miR-213, novel-miR-230, novel-miR-263, novel-miR-272, novel-miR-276) (Table 3; Additional file 9: Table S8). To gain further insights, we identified targets of these predicted siRNAs (Additional file 9: Table S8; siRNA targets sheet). Altogether, 3441 targets were predicted using psRNA target finder (Additional file 10: Table S9; B2G analysis sheet). Further, GO analysis of targets showed that off 5918 GO terms, 1780 belongs to biological processes, 2210 terms included in molecular function category, whereas 1919 GO terms categorised in cellular components (Additional file 10: Table S9). All GO terms were further subjected to KEGG pathway analysis for functional reconstruction of targets. Altogether, we obtained 103 enriched pathways from our KEGG analysis (Additional file 10: Table S9; KEGG pathways sheet). It was also observed that siRNA target genes were enriched in starch and sucrose, fructose and mannose, purine and pyrimidine as well as in several amino acid metabolism (Additional file 10: Table S9; KEGG analysis sheet; grey highlighted).

Interestingly, two of the phased siRNAs from *StTm2* and *StRGA4* found to target key GA metabolic genes *StGA2ox1* (Additional file 9: Table S8, line 862 of siRNA target sheet and Additional file 10: Table S9, line 2330 of B2G analysis sheet; yellow highlighted) and *StGA3ox1* (Additional file 9: Table S8, line 4443 of siRNA target sheet and Additional file 10: Table S9, line 443 of B2G analysis sheet; yellow highlighted), respectively. Apart from this, many genes involved in various other hormones (auxin, CK, ABA and ethylene) transport, metabolism and signalling were also identified as targets of siRNAs (Additional file 10: Table S9). Different target genes of siRNAs also included calcium signalling related genes (*CDPKs* and *calmodulin-* and *calcineurin- binding proteins*) (Additional file 10: Table S9, B2G analysis sheet; green highlighted), and cell-cycle and cell-division associated genes (*cyclin C5, cyclin D4/D6* and *cyclin-dependent kinases/inhibitors*) (Additional file 10: Table S9, B2G analysis sheet; brown highlighted). Additionally, genes encoding for different *homeobox TFs, F-box proteins, early flowering 3, Dof zinc finger protein (StCDF4), POTH1, phloem mobile RNA binding protein (StPTB1),* and *zinc/ring finger proteins* were found to be siRNA targets (Additional file 10: Table S9, B2G analysis sheet; blue highlighted).

### Validation of exonic region *TAS-like* loci and detection of putative phased siRNAs

**Fig. 8:**
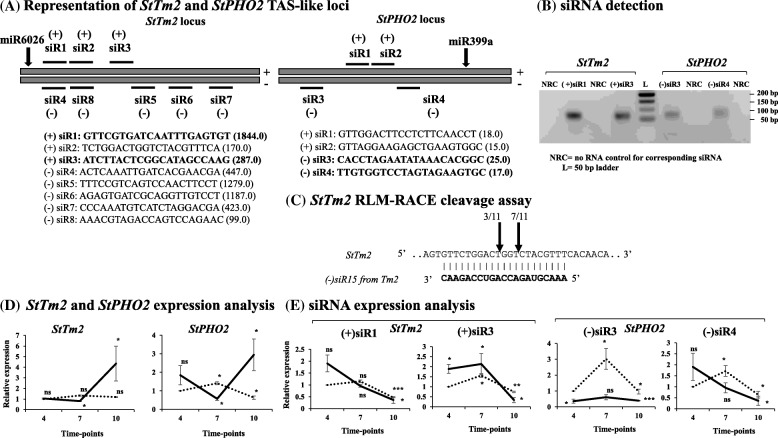
**a** Graphical representation of *StTm2* and *StPHO2 TAS-like* loci and the most abundant siRNAs generated from these two loci are shown. Phased siRNAs predicted to be generated from *StTm2* and *StPHO2*. Eight siRNAs off 18, whereas 4 siRNAs off 8, with higher abundance values are shown for both loci, respectively. Black arrows pointing downwards represent predicted miRNA cleavage site in each locus. **b** Detection of two phased siRNAs (highlightd in bold in panel (**a**) on sense strand of *StTm2* ([+]siR1 and [+]siR3) and two from antisense strand of *StPHO2* ([−]siR3 and [−]siR4) by RT-PCR. **c** 5′ RLM-RACE assay confirms the cleavage of *StTm2* by one of the siRNAs generated from itself i.e. (−)siR15. The siRNA cleavage frequency is also shown by downward arrows in black (10/11). Alignment between mature miRNA (bold) and target gene sequence is shown. **d** Expression analysis of *StTm2* and *StPHO2* at 4, 7 and 10 d time-points under LD vs SD photoperiod conditions. *EIF3e* was used as a reference gene for normalization. The expression level of respective target gene at LD4 was considered as 1 for measurement of relative expression at other time-points. Error bars represent standard error of means from two biological replicates. **e** Expression analysis for two phased siRNAs generated from *StTm2* ([+]siR1 and [+]siR3) and two from *StPHO2* ([−]siR3 and [−]siR4) loci at 4, 7 and 10 d time-points under LD vs SD photoperiod conditions. *U6* was used as a reference gene for normalization. The expression level of respective siRNA at LD4 was considered as 1 for measurement of relative expression at other time-points. Error bars represent standard error of means from two biological replicates. For graphs, a dotted line represents LD condition, whereas a thick line represents SD condition. Asterisks (one, two and three) indicate significant differences at *p* < 0.05, *p* < 0.01, *p* < 0.001, respectively, using a Student’s t-test. ns = not significant at *p* < 0.05

Two putative *TAS-like* loci (Chr09:15393324..15393575 and Chr02:33977738..33977989), that were predicted to be present within the genic regions of *StTm2* (predicted target of miR6026-3p) and *StPHO2* (predicted target of miR399a), respectively, were chosen for further analysis based on the targets of phased siRNAs they produce (Table S9). The partial transcript sequences of these two genes were detected in stolon samples through real-time PCR using primers on either side of predicted cleavage site (Fig. 8b). Subsequently, two phased siRNAs generated each from *StTm2* (2 off 18 siRNAs) and *StPHO2* (2 off 8 siRNAs) transcripts were also detected in stolon samples (Fig. 8b; Additional file 1: Figure S5) and were sequence confirmed. Further, a modified 5′ RLM-RACE confirmed *StTm2* transcript as a target of miR6026-3p with a very low frequency (1/11) (Fig. 6d). Interestingly, we observed that one of the siRNAs generated from *StTm2* locus, i.e. (−) siR8, cleaved its own locus with 10 of 11 cleavage frequency (Fig. 8c; Additional file 1: Figure S5), which might generate secondary siRNAs from *StTm2* locus. Since predicted cleavage site of miR6026-3p on *StTm2* locus is only 65 bp upstream to (−) siR8 generation/cleavage site, this could be the reason why the frequency of cleavage by miR6026 was very low (1/11) on this locus. From these results, it appeared that the efficiency of cleavage by (−) siR8 is higher (10/11) than that of miR6026-3p (1/11). Sequencing results for RACE cloning are shown in Additional file 1: Figure S6.

### Time-course expression analysis of genic *TAS-like* loci and their siRNAs

To investigate if photoperiod has any influence on the expression of *StTm2* and *StPHO2,* qRT-PCR assays were performed. *StTm2* showed a significant reduction in transcript abundance at SD7, whereas its transcript level was significantly enhanced at SD10 time point, when compared to LD4 (Fig. 8d). A significant increase in expression level was observed for *StPHO2* at LD7 and SD10 time points, whereas its transcript level remained significantly low at SD7 and LD10 time points compared to LD4 (Fig. 8d). Two siRNAs generated from *StTm2* (+siR1 and + siR3) and two from *StPHO2* (−siR3 and -siR4) *TAS*-like loci were selected for qRT-PCR analysis (Fig. 8e). From *StTm2*, both siRNAs (+siR1 and + siR3) showed a significant reduction under LD10 and SD10 time points compared to LD4. However, +siR3 levels were significantly high under SD4, LD7 and SD7 time points when compared to LD4 (Fig. 8e). From *StPHO2*, −siR3 exhibited a significant reduction in its expression under SD at 4 and 10 d time points, whereas a significant increase in its expression was observed at LD7 and LD10 compared to LD4 (Fig. 8e). -siR4 exhibited a significantly high expression at LD7 time point, but its expression was found to be significantly low at both LD and SD 10d time points compared to LD4 (Fig. 8e).

### Conserved *TAS* loci in potato

**Fig. 9:**
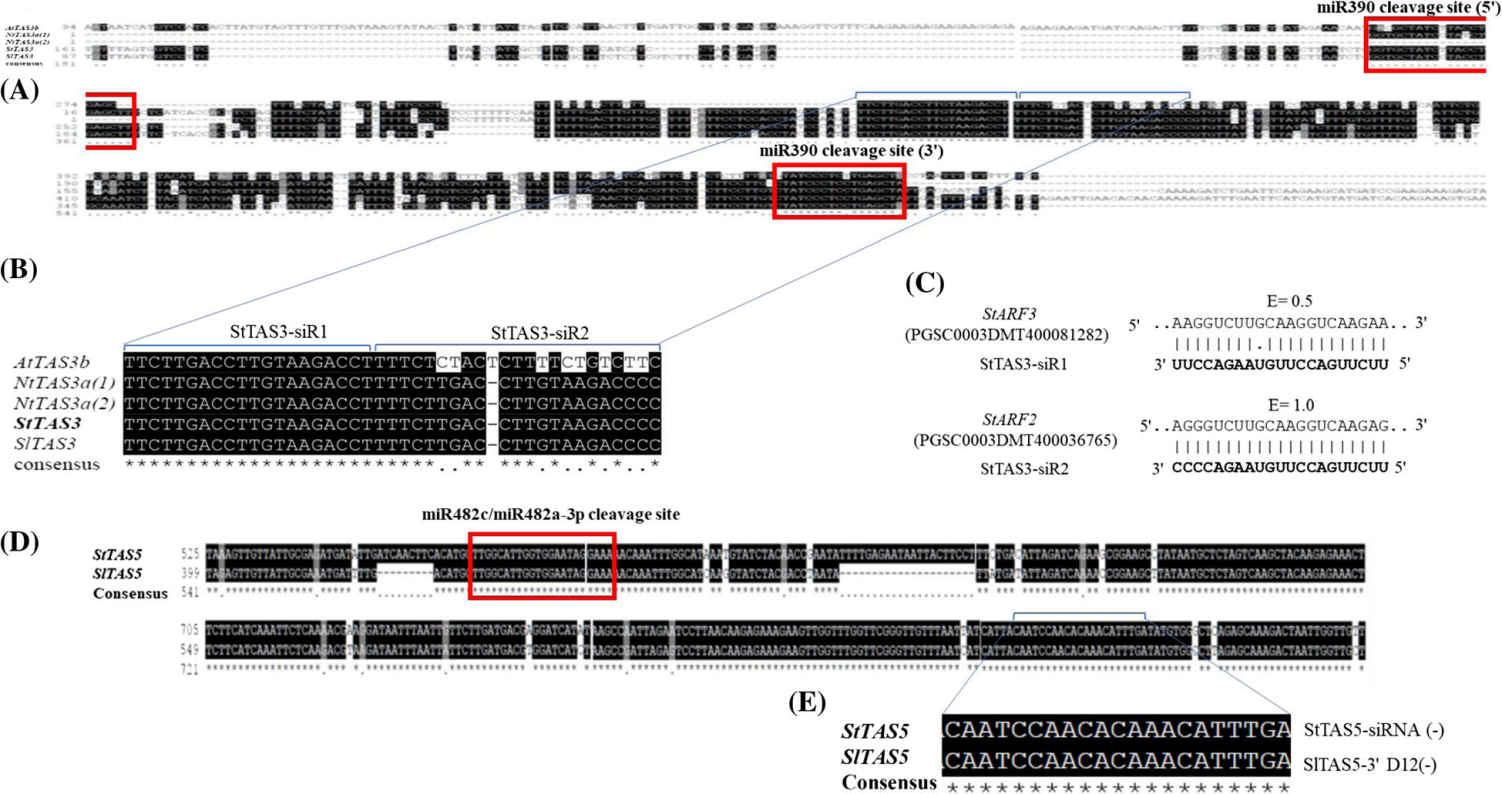
**a** Alignment of *TAS3* transcripts from *Arabidopsis thaliana AtTAS3b*, *Nicotiana tabacum NtTAS3a (1)* and *NtTAS3a (2)*, *Solanum tuberosum StTAS3* and *Solanum lycopersicum SlTAS3*. miR390 target sites (5′ and 3′) on all *TAS3* transcripts are highlighted with red boxes. **b** Magnified view of the alignment of *TAS3* transcripts from above mentioned plant species. StTAS3-siR1, with an abundance value of 134 and StTAS3-siR2, with an abundance value of 148, showed a close conservation across chosen plant species. **c** psRNATarget prediction shows that StTAS3-siR1 cleaves *Auxin Response Factor StARF3,* whereas StTAS3-siR2 cleaves *StARF2* with a cleavage expectancy of E = 0.5 and E = 1.0, respectively. Accessions: *StARF3,* PGSC0003DMT400081282; *StARF2,* PGSC0003DMT400036765. **d** Alignment of *Solanum tuberosum StTAS5* transcript with that of *Solanum lycopersicum SlTAS5* transcript. miR482c/miR482a-3p target site is highlighted in red box. **e** Magnified view of *TAS5* transcripts alignment from potato and tomato. StTAS5-siRNA (−), with an abundance value of 1734, showed a high conservation with tomato SlTAS5–3′ D12(−)

In our analysis, one *TAS* locus (Chr01:37276576..37276827, hereafter referred as *StTAS3*) fulfilled the *TAS3* criteria as described in Xia et al. [4] (Additional file 8: Table S7; line 21; red highlighted). This locus was present in the genic region of a hypothetical protein (PGSC0003DMT400034044) and contains two miR390 cleavage sites. When *StTAS3* transcript was aligned to *Arabidopsis thaliana AtTAS3b*, *Nicotiana tabacum NtTAS3a* (1) and *NtTAS3a* (2), and *Solanum lycopersicum SlTAS3*, a close conservation across the plant species was observed. Moreover, two siRNAs regions StTAS3-siR1 (abundance value 134) and StTAS3-siR2 (abundance value 148) showed a high conservation in all the above mentioned plant species. These siRNAs were predicted to cleave *StARF3* and *StARF2* transcripts with expectancy values of 0.5 and 1.0, respectively (Fig. 9). Thus, *TAS* identified in our analysis appears to be a potential *StTAS3* locus.

Apart from *StTAS3*, we also found *TAS5* locus in potato (Chr06:5518510..5518761, hereafter referred as *StTAS5*), which reside in the NBS-coding resistance gene (PGSC0003DMT400022440), and this locus shared a close conservation with *SlTAS5* from tomato (Additional file 8: Table S7; line 133; red highlighted). Moreover, the target site of miR482c/miR482a-3p on these loci was conserved. Similarly, StTAS5-siRNA[−], with abundance value of 1734, displayed a high conservation with a tomato SlTAS5–3′ D12(−) (Fig. 9).

## Discussion

### Photoperiod influences miRNA expression during early stages of stolon-to-tuber transitions

In tuberization, the stolon acts as a focal point that coordinates several mobile signals, transcription factors (TFs), hormones [20, 21], and involves interaction between environmental, biochemical, and genetic factors [17]. At the onset of short-day tuber induction, elongating stolon ceases its growth and undergoes numerous changes. At the stolon tip, the fate of cell division changes from longitudinal to transverse, followed by a number of random cell divisions at the sub-apical region resulting in swelling of stolon, which eventually gives rise to a mature tuber [18, 19]. The majority of plant miRNAs control developmental decisions of cell differentiation or organ patterning by targeting multiple families of transcription factors [39]. It is plausible that the dynamic changes at the stolon tip could be governed by many molecular factors including sRNAs. We focussed on early stages of stolon development because at this stage key molecular players are likely to be active in regulating and fine tuning the tuber formation process. No previous report has described the role of sRNAs involved in this transition process under photoperiodic conditions. To decipher this, we performed a sRNA profiling from stolons of Andigena plants at three early stages (4, 7 and 10 d) of stolon-to-tuber transitions under LD verses SD photoperiod conditions. We observed that 7 out of 324 conserved and 12 out of 311 novel miRNAs exhibited differential expression under SD at either 4 or 10 d time point compared to respective LD conditions. Five conserved miRNAs (miR477a-5p, miR477b-5p, miR319-3p, miR8006-5p and miR479) as well as three novel miRNAs (n-miR-147, n-miR-139 and n-miR-40) showed a significant increase in their expression levels under SD conditions at 4, 7 or 10 d time points compared to LD4 (Fig. 3). In contrast, a distinct reduction in the expression of several conserved/novel miRNAs (miR482d-3p, miR99g-3p, n-miR-139, n-miR-302, n-miR-221, n-miR-93 and n-miR-276) were observed under SD conditions at 4, 7 or 10 d time points compared to LD4 (Fig. 3). Interestingly, one novel miRNA, n-miR-206, exhibited significantly higher expression under SD conditions than LD conditions at all three time points tested (Fig. 3). These findings suggest that the early developmental stages could be under the control of key sRNAs in a photoperiod dependent manner.

Previously, the expression of miR156 and miR172 were shown to be induced in stolons at 14 d time point under SD conditions and both were implicated in tuber development [40, 41]. Wu et al. demonstrated that a pooideae-specific miR5200 is expressed at high level under short-days and drastically reduced under long-days and mediates post-transcriptional modulation of Flowering Locus T to govern floral transitions in Brachypodium [42]. Another microRNA (miR163) was shown to be highly induced by red/blue/white light whereas its target *AtPXMT1* (methyl transferase) was down-regulated during seedling de-etiolation and germination in Arabidopsis [43]. Several other studies have also identified blue/UV-B light responsive miRNAs in Arabidopsis [44], wheat [45] and *Brassica rapa* [46]. From our study, it appears that early stage of stolon development (4 d) is crucial since 17 out of 19 differentially expressed miRNAs (~ 90%) were found at this time point compared to our observations at 7 and 10 d time points (Table 2). This suggests that these miRNAs and their putative targets might play important roles in regulating the early stages of the stolon-to-tuber transition. Only future investigation can unravel the process.

### MiRNA targets and their potential role in tuber development

Previous studies have shown that tuber development is regulated by several hormones [18, 19, 47, 48]. It is not unlikely that the early stages of stolon-to-tuber transition process are governed by any of these hormones considering the fact that the stolon tip undergoes rapid cell division and differentiation process. Our analysis identified many interesting hormone metabolism related genes as putative targets of conserved/novel miRNAs (Additional file 6: Table S5, Additional file 7: Table S6). Several targets of miRNAs exhibited differential expression under SD/LD photoperiodic conditions (Fig. 7), suggesting that miRNA mediated regulation of target genes could be crucial to fine tune the early stages of stolon-to-tuber transitions. DELLA [49] and GAMYB [50, 51] have an established role in mediating GA dependent promotion of flowering in Arabidopsis. Expression analysis showed that *StGAMYB* is differentially expressed during early stages of tuber development (Fig. 7), pointing towards the role of GA signaling components during stolon transitions. Moreover, by 5′ RLM-RACE, we have confirmed that *StGAMYB* TF as a target of miR319b (Fig. 5c). GRAS TFs are involved in root radial patterning and root growth [52]. GRAS domain, an important domain among GRAS TFs, contain residues for protein-protein interaction as well as for DNA binding [53]. In our analysis, *StGRAS* TF was found to be a target of miR479 and the presence of cleavage site within the GRAS domain (Fig. 5b) indicated a diverse role of miR479 in developmental processes. Complementary relationship between miR319-3p and *StTCP2/4* TFs at early stages of stolon development under SD/LD condition (Figs. 3-4, Fig. 7) indicates a possible role in cell-division and proliferation at the stolon tip, necessary for stolon growth. TCP TFs are known to be involved in various hormone metabolism [54, 55]. TCP2’s function as a transcriptional activator down-stream to CRYPTOCHROME 1 (CRY1) photo-sensory signaling [56] and the role of TCP4 in maintaining cell number as well as cell proliferation during leaf development has been demonstrated [57].

Cho et al. have shown that polypyrimidine tract-binding proteins in potato (StPTB1 and − 6) bind to *StBEL5* mRNA to facilitate transport of its mRNA from leaves to stolons under tuber inductive conditions [24]. In this indirect process, StPTBs function as positive regulators of tuberization by enhancing *StBEL5* mRNA levels in stolons [58]. The psRNA target prediction analysis suggests that *StPTB6* mRNA could be the target of miR156e (Additional file 6: Table S5, Additional file 7: Table S6; line 2087 of conserved miRNAs and targets sheet and line 1316 of target genes annotation sheet; yellow highlighted). Further, the transcript levels of *StPTB6* were increased under SD conditions at 4, 7 and 10 d time-point compared to LD time-points (Fig. 7), suggesting a new mode for regulation of *StPTB6* by miR156e during tuber development. Two close homologs of StBEL5 in potato; StBEL11 and StBEL29; are reported to function as phloem mobile RNA signals like *StBEL5*, but they act as repressors of tuber yield [59]. Through degradome analysis, it was found that *StBEL29* transcript is the target of a conserved miRNA (miR172h) in cold-stored potato tubers [60]. Moreover, novel miRNAs, such as n-miR-221 and n-miR-230, have been shown to target histone-lysine N-methyltransferases (ASHR3 and ATX4-like, respectively). Differential expression of these target genes (*StASHR3* and *StSWI3*) during early stages of stolon transitions under SD/LD conditions (Fig. 7), suggests a possible epigenetic regulation of this transition process.

### Phased siRNAs and their potential targets in tuberization

The phased siRNA (21–22 or 24 nt) family functions in either *cis* - (phasiRNA) or *trans* - (tasiRNA) manner [2] and is involved in various developmental and stress responses in plants [6–12]. No previous study has reported the potential role of phased siRNAs in potato development. Only a few studies have predicted the repertories of phased siRNAs in the Solanaceae family, mainly involved in defense responses [61–63]. In this study, using the TA-SI prediction tool, we identified 830 putative *TAS-like* loci from the potato genome, which can give rise to either phasi or tasiRNAs. Our analysis has identified two conserved *TAS* loci (*StTAS3* and *StTAS5*) from stolon samples in potato (Fig. 9). This suggests the reliability and accuracy of small RNA analysis and *TAS* loci prediction tool used in this study. Off 830 *TAS-like* loci, 24 were found to be cleaved by either conserved or novel miRNAs (Table 3) that generate 190 phased siRNAs (Additional file 9: Table S8; TAS-loci sheets). The target prediction tool yielded 3441 unique targets having diverse functions (Additional file 10: Table S9; B2G analysis sheet) indicating that some of these siRNAs might function as tasiRNAs.

During the stolon-to-tuber formation process, stolon tips function as a strong sink for starch and storage protein accumulation and as anticipated, siRNA target genes were enriched in starch/sucrose metabolism as well as protein biosynthesis. Several studies have implicated the role of calcium and calcium-dependent protein kinases (CDPKs) in tuber development [25, 26, 64]. In our study, we found that a few genes encoding for *CDPKs* and *calmodulin/calcineurin binding proteins* (Additional file 10: Table S9, B2G analysis sheet; green highlighted), as well as several cell-cycle and cell-division associated genes (*cyclin C5, cyclin D4/D6* and *cyclin-dependent kinases/inhibitors*) served as siRNA targets (Additional file 10: Table S9, B2G analysis sheet; brown highlighted). It is believable that these target genes could be expressed in a spatio-temporal manner at the stolon tip considering the dynamic changes during the process of stolon-to-tuber transition. Additionally, target genes implicated in tuberization such as *StPTB1* [24], *StCDF4* [34] and a homeobox TF, *POTH1* [25] have been identified as siRNA targets (Additional file 10: Table S9, B2G analysis sheet; blue highlighted). Several other genes encoding *F-box proteins, early flowering 3,* and *zinc/ring finger proteins* were also found to be the targets of siRNAs (Additional file 10: Table S9, B2G analysis sheet; blue highlighted). Interestingly, one phased siRNA from *StTm2* and other one from *StRGA4* locus were found to cleave the mRNA of GA metabolic genes *StGA2ox1* (Additional file 9: Tables S8, line 862 of siRNA target sheet and Additional file 10: Table S9, line 2330 of B2G analysis sheet; yellow highlighted) and *StGA3ox1*, respectively (Additional file 9: Table S8, line 4443 of siRNA target sheet and Additional file 10: Table S9, line 443 of B2G analysis sheet; yellow highlighted). In the process of tuberization, GA levels goes down due to the increased levels of a GA catabolic gene (*StGA2ox1*) [47] and reduced levels of biosynthetic genes (*StGA3ox1* and *StGA20ox1*) [25, 65], indicating a new layer of regulation in potato development by phased siRNAs. Further, the validation of two *TAS-like* loci (*StTm2* and *StPHO2*) confirm that many of the phased siRNAs generated from these loci could eventually target various genes **(**e.g. *squamosa promoter-binding-like protein 7, pectin esterase, sugar transporter ERD6-like 5, protein phosphatase 2c, LRR receptor, beta-glucosidase, aquaporin protein, ubiquitin-conjugating enzyme*, *protein argonaute 7, glycosyltransferase 7, ethylene responsive E3 ubiquitin-protein ligase XBAT35* and *various transcription factors*) (Additional file 10: Table S9; grey highlighted) and they could function as tasiRNAs.

## Conclusions

In this study, we have identified 7 conserved and 12 novel miRNAs to be differentially expressed in early stages of stolon-to-tuber development in a photoperiod dependent manner. Select putative targets of these miRNAs also exhibited differential expression during early stolon stages under SD or LD conditions. Out of 830 *TAS-like* loci predicted in our analysis, two conserved *TAS* loci, such as, *StTAS3* and *StTAS5*, were identified in this study. Apart from this, we identified 24 *TAS*-like loci that would generate 190 unique phased siRNAs. Target identification of phased siRNAs showed that several putative tuberization genes were targeted by them. *StTm2 TAS* locus was validated by 5′ RLM-RACE for generation of phased siRNAs. One of the siRNA from this locus was predicted to target *StGA2ox1* and we speculate that it could be an important candidate in regulation of the early stages of stolon-to-tuber transition in potato. Future experiments are needed to establish the mechanistic link between miRNAs/phased siRNAs and their putative targets during early stages of tuber development.

## Additional files

Additional file 1: **Figure S1.** Early stolon transitions in *S. tuberosum* ssp. *andigena* (7540) with respect to short days (SDs). **Figure S2.** MA scatter plot analysis showing Log 2-fold change (y-axis) of pairwise comparisons for conserved and novel microRNAs respectively- LD4 vs SD4 (A & B), LD7 vs SD7 (C & D) and LD10 vs SD10 (E & F). **Figure S3.** Heat map clustering for top 30 representative conserved miRNAs (A) and novel miRNAs (B) from all 12 LD and SD stolon libraries. **Figure S4.** Predicted secondary structures of three novel and three conserved miRNAs as determined using UEA small RNA workbench are shown. Mature miRNA sequences are highlighted in green. **Figure S5.** Representation of *StTm2* and *StPHO2 TAS-like* loci. siRNAs generated from these loci due their cleavage by stu-miR6026-3p and stu-miR399i-3p, respectively. **Figure S6.** Sequencing results for RLM-RACE cloning of *StARF10*, *StGRAS*, *StGAMYB* and *StTm2* in pGEMT vector are given. (DOC 515 kb)

Additional file 2: **Table S1.** List of primers (XLSX 12 kb)

Additional file 3: **Table S2.** List of conserved and novel miRNAs (XLSX 104 kb)

Additional file 4: **Table S3.** DE analysis- Conserved miRNAs (XLSX 112 kb)

Additional file 5: **Table S4.** DE analysis- Novel miRNAs (XLSX 113 kb)

Additional file 6: **Table S5.** psRNA targets- Conserved and Novel miRNAs (XLSX 217 kb)

Additional file 7: **Table S6.** B2G annotation for miRNA targets (XLSX 210 kb)

Additional file 8: **Table S7.** *TAS* loci prediction (XLSX 104 kb)

Additional file 9: **Table S8** siRNAs from *TAS* and target prediction (XLSX 377 kb)

Additional file 10: **Table S9.** B2G annotation for siRNA targets (XLSX 489 kb)

## Abbreviations

CK: Cytokinin
FP: Forward primer
GA: Gibberellin
GO: Gene ontology
LD: Long day
miRNAs: MicroRNAs
nt: Nucleotide
phased siRNAs: Phased short-interfering RNAs
POTH1: Potato class-I KNOX gene
qRT-PCR: Quantitative reverse transcriptase polymerase chain reaction
RLM-RACE: RNA-ligase mediated rapid amplification of cDNA ends
RP: Reverse primer
SD: Short day
sRNAs: Small RNAs
StASHR3: Potato histone-lysine N-methyltransferase
StBEL5/11/29: Potato BEL1-like TFs 5/11/29
StCDF4: Potato cyclin DOF family TF 4
StCDPK: Potato calcium-dependent protein kinase
StDELLA: Potato GA signaling repressor
StGAMYB: Potato GA-induced MYB family TF
StPTB1/6: Potato poly-pyrimidine tract-binding proteins 1/6
StSWI3: Potato Histone-lysine N-methyltransferase
StTCP2/4: Potato TCP TFs 2/4
tasiRNAs: Trans-acting short-interfering RNAs
TFs: Transcription factors
