# Supplementary material for: Genome-wide transcriptome analysis reveals small RNA profiles involved in early stages of stolon-to-tuber transitions in potato under photoperiodic conditions
Source: BMC Plant Biol. 2018 Nov 16;18:284. doi: 10.1186/s12870-018-1501-4 (PMC6238349; doi:10.1186/s12870-018-1501-4)
Supplement: Supplementary file 1 — Figure S1. Early stolon transitions in S. tuberosum ssp. andigena (7540) with respect to short days (SDs). Figure S2. MA scatter plot analysis showing Log 2-fold change (y-axis) of pairwise comparisons for conserved and novel microRNAs respectively- LD4 vs SD4 (A & B), LD7 vs SD7 (C & D) and LD10 vs SD10 (E & F). Figure S3. Heat map clustering for top 30 representative conserved miRNAs (A) and novel miRNAs (B) from all 12 LD and SD stolon libraries. Figure S4. Predicted secondary structures of three novel and three conserved miRNAs as determined using UEA small RNA workbench are shown. Mature miRNA sequences are highlighted in green. Figure S5. Representation of StTm2 and StPHO2 TAS-like loci. siRNAs generated from these loci due their cleavage by stu-miR6026-3p and stu-miR399i-3p, respectively. Figure S6. Sequencing results for RLM-RACE cloning of StARF10, StGRAS, StGAMYB and StTm2 in pGEMT vector are given. (DOC 515 kb) [file 12870_2018_1501_MOESM1_ESM.doc]

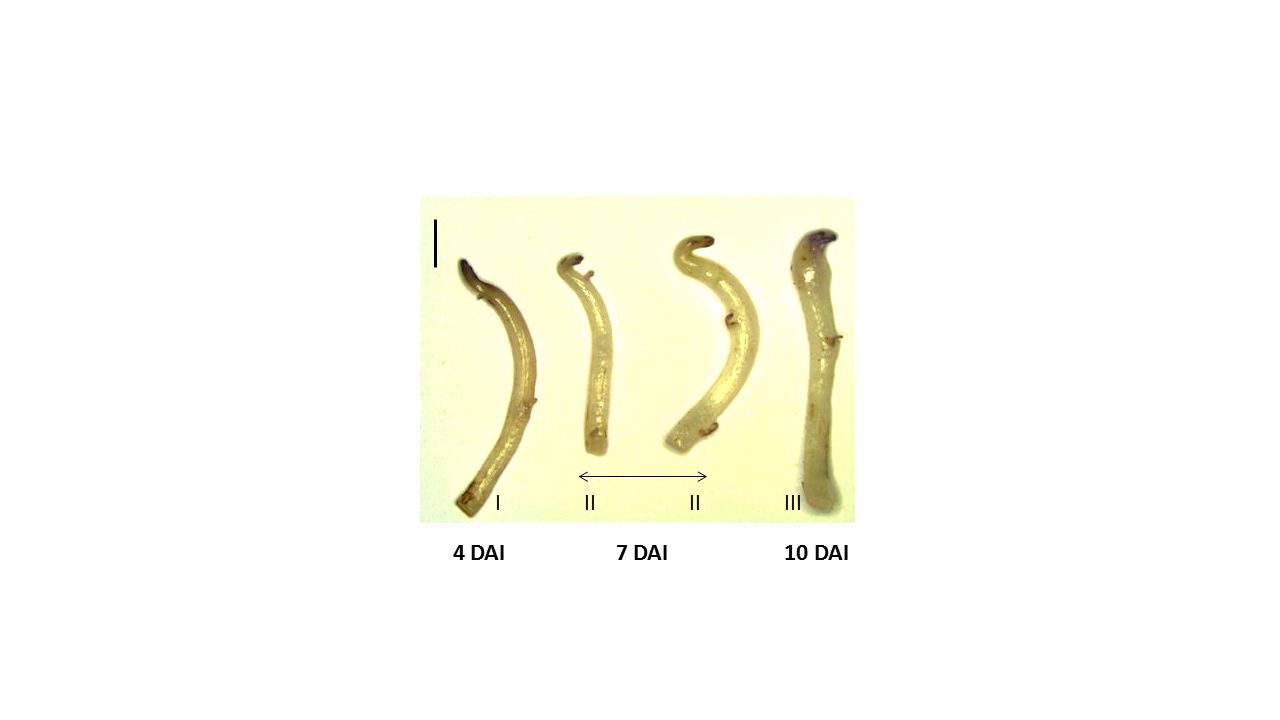


**Additional file: Fig. S1.** Early stolon transitions in *S. tuberosum* ssp. *andigena* (7540) with respect to short days (SDs). Under SDs, a stolon passes through different transitions to form a tuber. Panels (I), (II) and (III) represent different stages of stolon development at 4, 7 and 10 days during short/long day after induction (DAI), respectively. Under SDs, most stolons at 7 DAI (II) are characterised by a distinct apical hook; whereas stolons at 10 DAI (III) are characterised by swelling at the apical region. Bar is 2 mm.

**
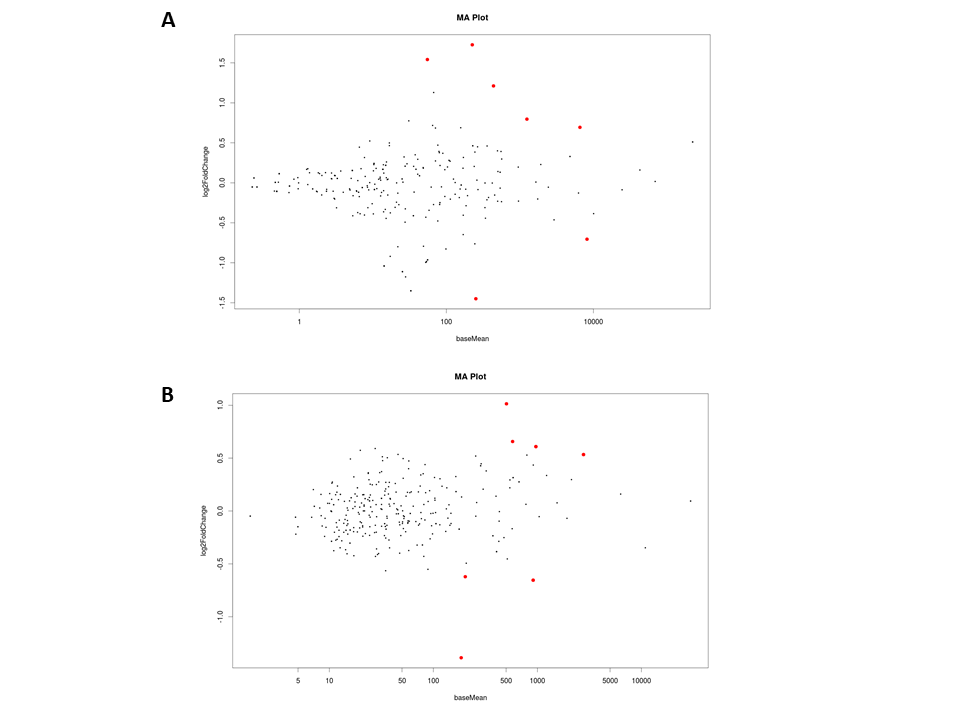
**

**
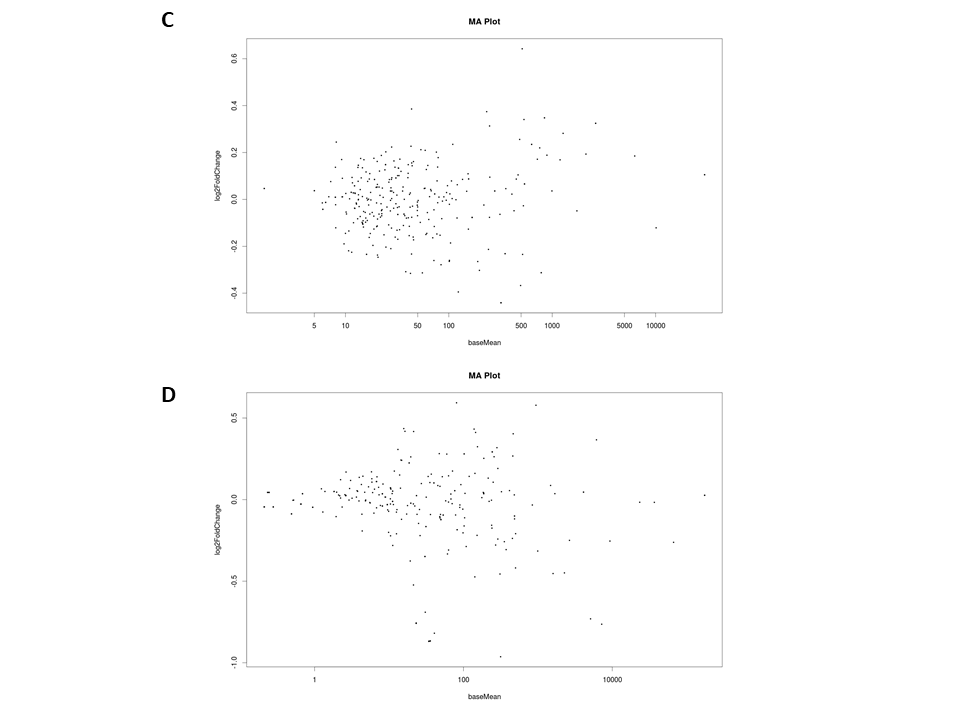
**

**
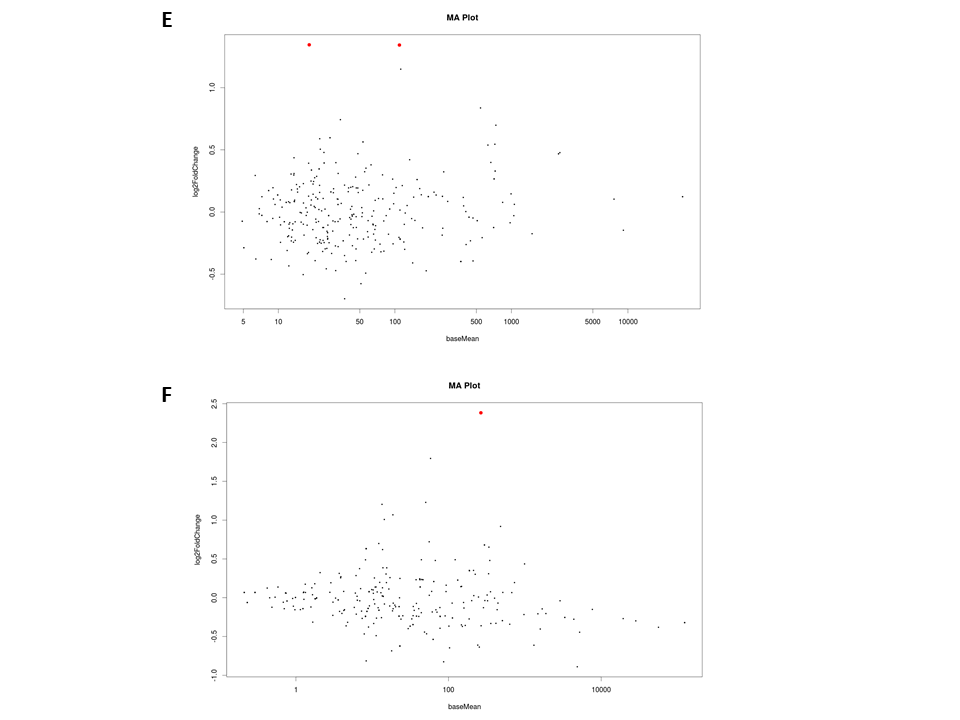
**

**Additional file: Fig. S2.** MA scatter plot analysis showing Log 2-fold change (y-axis) of pairwise comparisons for conserved and novel microRNAs respectively- LD4 vs SD4 (A & B), LD7 vs SD7 (C & D) and LD10 vs SD10 (E & F). Differentially expressed miRNAs in each LD vs SD comparisons are highlighted with dark red dot.

**
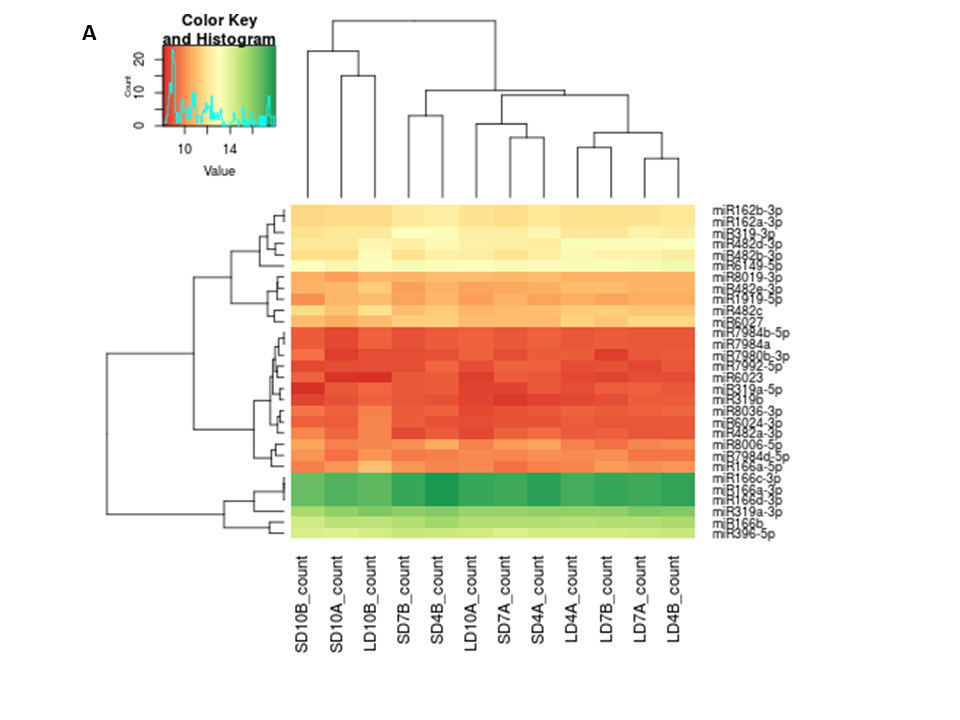
**

**
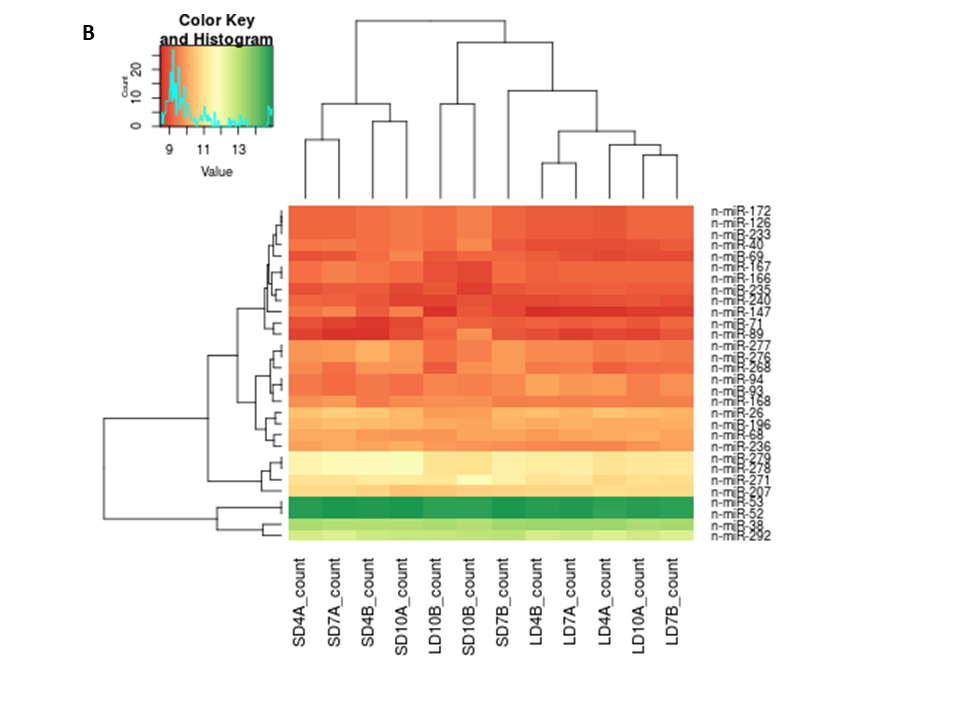
**

**Additional file: Fig. S3.** Heat map clustering for top 30 representative conserved miRNAs (A) and novel miRNAs (B) from all 12 LD and SD stolon libraries.


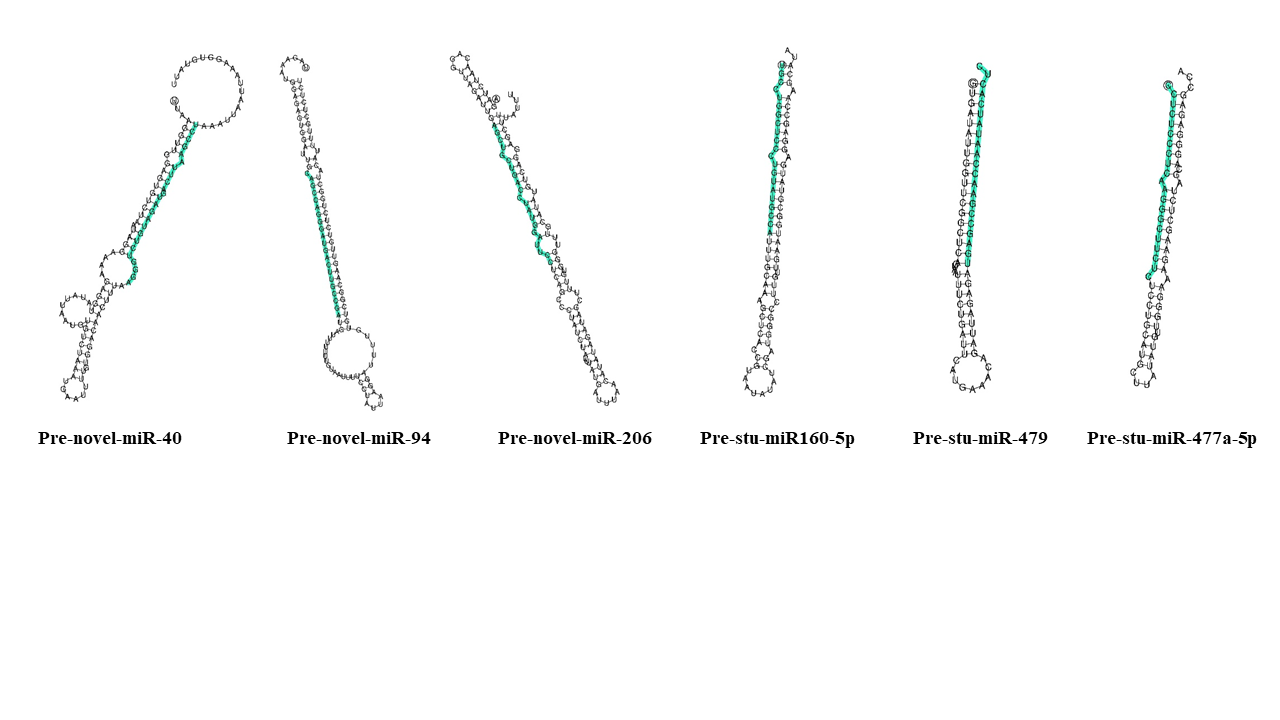


**Additional file: Fig. S4.** Predicted secondary structures of three novel and three conserved miRNAs as determined using UEA small RNA workbench are shown. Mature miRNA sequences are highlighted in green.

**(A) *StTm2* locus (>ST4.03ch09-Start:15393324-End:15393575)** Targeted by stu-miR6026-3p

**Locus sequence length**: 252 bp (1……252 bp)

GGTCTGG**GGAAAACAACTXCTTGCCAAGAA**ACTTTATAGGCTCGTTCGTGATCAATTTGAGTGTTCTGGACTGGTCTACGTTTCACAACAGCCAAGAGCGGGAGAAATCTTACTCGGCATAGCCAAGGAAGTTGGACTGACGGAAAAGAAAAGGAAGGAACATTTGGAGGACAACCTGCGATCACTCTTGAAAATAAAAAGGTATGTTATCGTCCTAGATGACATTTGGGATGTTGAAATTTGGGATGATCTG

(+) siR1: GTTCGTGATCAATTTGAGTGT (1844.0) Location: 43-63 bp= 21 bp

(+) siR2: TCTGGACTGGTCTACGTTTCA (170.0) Location: 64-84 bp= 21 bp

(+) siR3: ATCTTACTCGGCATAGCCAAG (287.0) Location: 106-126 bp= 21 bp

(-) siR4: ACTCAAATTGATCACGAACGA (447.0) Location: 41-61 bp= 21 bp

(-) siR5: TTTCCGTCAGTCCAACTTCCT (1279.0) Location: 125-145 bp= 21 bp

(-) siR6: AGAGTGATCGCAGGTTGTCCT (1187.0) Location: 167-187 bp= 21 bp

(-) siR7: CCCAAATGTCATCTAGGACGA (423.0) Location: 209-229 bp= 21 bp

(-) siR8: AAACGTAGACCAGTCCAGAAC (99.0) Location: 62-82 bp=21bp (targets *Tm2*)

(+) siR9: GCCAAGAAACTTTATAGGCTC (91.0) Location: 22-42 bp= 21 bp

(+) siR10: CAACAGCCAAGAGCGGGAGAA (47.0) Location: 85-105 bp= 21 bp

(+) siR11: AAAAGGAAGGAACATTTGGAG (23.0) Location: 148-168 bp= 21 bp

(+) siR12: GGTCTGGGGAAAACAACTCTT (16.0) Location: 1-21 bp= 21 bp

(+) siR13: GACAACCTGCGATCACTCTTG (8.0) Location: 169-189 bp= 21 bp (GA2ox1)

(+) siR14: GAAGTTGGACTGACGGAAAAG (7.0) Location: 127-147 bp= 21 bp

(+) siR15: AAAATAAAAAGGTATGTTATC (7.0) Location: 190-210 bp= 21 bp

(-) siR16: CTCCCGCTCTTGGCTGTTGTG (50.0) Location: 83-103 bp= 21 bp

(-) siR17: CCAAATGTTCCTTCCTTTTCT (13.0) Location: 146-166 bp= 21 bp

(-) siR18: TGGCTATGCCGAGTAAGATTT (2.0) Location: 104-124 bp= 21 bp

**(B) *StPHO2* Locus (>ST4.03ch02-Start:33977738-End:33977989)** Targeted by stu-miR399i-3p

**Locus sequence length**: 252 bp (1……252 bp)

CGAGGTTTGGTGGTTCTTTGCCGTGTTTATATTCTAGGTGTCATCTTCCTTCGTTGGTAAAAAGTTGATCCATATTTCTGGCAAGTTGGACTTCCTCTTCAACCTGTTAGGAAGAGCTGAAGTGGCACTTCTACTAGGACCACAATTAATTGGAACTTTCTTCTCCACGCAACCCTTCTAGTGGATTTCGTTTCT**CTGGGCAAATXCTCCTTTGGCA**TTTCGGAGTTGTTAGACTCCACCACCGTTTGTCTTT

(+) siR1: GTTGGACTTCCTCTTCAACCT (18.0) Location: 85-105 bp= 21 bp

(+) siR2: GTTAGGAAGAGCTGAAGTGGC (15.0) Location: 106-126 bp= 21 bp

(-) siR3: CACCTAGAATATAAACACGGC (25.0) Location: 20-40 bp= 21 bp

(-) siR4: TTGTGGTCCTAGTAGAAGTGC (17.0) Location: 125-145 bp= 21 bp

(+) siR5: CGAGGTTTGGTGGTTCTTTGC (6.0) Location: 1-21 bp= 21 bp

(+) siR6: TTGGCATTTCGGAGTTGTTAG (4.0) Location: 211-231 bp= 21 bp

(+) siR7: GTTTCTCTGGGCAAATCTCCT (2.0) Location: 190-210 bp= 21 bp

(-) siR8: CACTTCAGCTCTTCCTAACAG (2.0) Location: 104-124 bp= 21 bp

**Footnote**: Symbols ‘+’ before siRNA number means originating from sense strand, whereas that of ‘-’ means siRNA originating from antisense strand. miRNA target site in bold and yellow; Number in bracket after siRNA sequences represent abundance value for phased siRNAs. ‘X’ symbol in blue represents predicted miRNA cleavage site. miRNA alignment region is highlighted in bold and yellow. All siRNAs are predicted to be of 21 nt in length. From 18 siRNAs generated from *Tm2* locus, it was confirmed by RLM-RACE that (-) siR8 targets *Tm2* locus itself, whereas (+) siR13 was predicted to target *GA2ox1* transcript.

**Additional file: Fig. S5.** Representation of *StTm2* and *StPHO2* TAS-like loci. siRNAs generated from these loci due their cleavage by stu-miR6026-3p and stu-miR399i-3p, respectively.

1. ***StARF10***
2. *StARF10*-1

GAACACTGCGTTTGCTGGCTTTGATGAA**X**GAGCCAGGCATGCTCAATTTGGAGTACCTTTATTGGATCTTCACCTTAGCAACAACTTACCGTCCGGATTGCTACCACCAAGTTTCCAGCGTGTTGCAGC

1. *StARF10*-2

GAACACTGCGTTTGCTGGCTTTGATGAA**X**GAGCCAGGCATGCTCAATTTGGAGTACCTTTATTGGATCTTCACCTTAGCAACAACTTACCGTCCGGATTGCTACCACCAAGTTTCCAGCGTGTTGCAGC

1. *StARF10*-3

AACACGCTGGAAACTTGGTGGTAGCAATCCGGACGGTAAGTTGTTGCTAAGGTGAAGATCCAATAAAGGTACTCCAAATTGAGCATGCCTGGCTC**X**TTCATCAAAGCCAGCAAACGCAGTGTTC

1. *StARF10*-4

GTGATTGCTGCAACACGCTGGAAACTTGGTGGTAGCAATCCGGTGAGTAAGTTGTTGCTAAGGTGAAGATCCAATAAAGGTACTCCAAATTGAGCATGCCTGGCTC**X**TTCATCAAAGCCAGCAAACGCAGTGTTC

1. *StARF10*-5

GAACACTGCGTTTGCTGGCTTTGATGAA**X**GAGCCAGGCATGCTCAATTTGGAGTACCTTTATTGGATCTTCACCTTAGCAACAACTTACCGTCCGGATTGCTACCACCAAGTTTCCAGCGTGTTGCAGC

1. *StARF10*-6

GTGATTGCTGCAACACGCTGGAAACTTGGTGGTAGCAATCCGGACGGTAAGTTGTTGCTAAGGTGAAGATCCAATAAAGGTACTCCAAATTGAGCATGCCTGGCTC**X**TCATCAAAGCCAGCAAACGCAGTGTTC

1. *StARF10*-7

GAACACTGCGTTTGCTGGCTTTGATGAA**X**GAGCCAGGCATGCTCAATTTGGAGTACCTTTATTGGATCTTCACCTTAGCAACAACTTACCGTCCGGATTGCTACCACCAAGTTTCCAGCGTGTTGCAG

1. ***StGRAS***
2. *StGRAS*-1

CGCGGATCCGAACACTGCGTTTGCTGGCTTTGATG**X**TTCGGCTCAAGGAATTGGCGTCCCCAAACGGCGCTACCAATATGGAGAGATTGGCCGCGCATTTCACTGACGCCTTGCAGGCGTTGCTCGACGGCGCTGCTTCCGGTACGTTACACG

1. *StGRAS*-2

CGTGTAACGTACCGGAAGCAGCGCCGTCGAGCAACGCCTGCAAGGCGTCAGTGAAATGCGCGGCCAATCTCTCCATATTGGTAGCGCCGTTTGGGGACGCCAATTCCTTGAGCCGAA**X**CATCAAAGCCAGCAAACGCAGTGTTC

1. *StGRAS*-3

CGTGTAACGTACCGGAAGCAGCGCCGTCGAGCAACGCCTGCAAGGCGTCAGTGAAATGCGCGGCCAATCTCTCCATATTGGTAGCGCCGTTTGGGGACGCCAATTCCTTGAGCCGAA**X**CATCAAAGCCAGCAAACGCAGTGTTC

1. *StGRAS*-4

GAACACTGCGTTTGCTGGCTTTGATG**X**TTCGGCTCAAGGAATTGGCGTCCCCAAACGGCGCTACCAATATGGAGAGATTGGCCGCGCATTTCACTGACGCCTTGCAGGCGTTGCTCGACGGCGCTGCTTCCGGTACGTTACACG

1. *StGRAS*-5

CGTGTAACGTACCGGAAGCAGCGCCGTCGAGCAACGCCTGCAAGGCGTCAGTGAAATGCGCGGCCAATCTCTCCATATTGGTAGCGCCGTTTGGGGACGCCAATTCCTTGAGCCGAA**X**CATCAAAGCCAGCAAACGCAGTGTTCGGATCCGCG

1. *StGRAS*-6

GAACACTGCGTTTGCTGGCTTTGATG**X**TTCGGCTCAAGGAATTGGCGTCCCCAAACGGCGCTACCAATATGGAGAGATTGGCCGCGCATTTCACTGACGCCTTGCAGGCGTTGCTCGACGGCGCTGCTTCCGGTACGTTACACG

1. *StGRAS*-7

CGTGTAACGTACCGGAAGCAGCGCCGTCGAGCAACGCCTGCAAGGCGTCAGTGAAATGCGCGGCCAATCTCTCCATATTGGTAGCGCCGTTTGGGGACGCCAATTCCTTGAGCCGAA**X**CATCAAAGCCAGCAAACGCAGTGTTCGGATCCGCG

1. *StGRAS*-8

CGCGGATCCGAACACTGCGTTTGCTGGCTTTGATG**X**TTCGGCTCAAGGAATTGGCGTCCCCAAACGGCGCTACCAATATGGAGAGATTGGCCGCGCATTTCACTGACGCCTTGCAGGCGTTGCTCGACGGCGCTGCTTCCGGTACGTTACACG

1. *StGRAS*-9

GAACACTGCGTTTGCTGGCTTTGATG**X**TTCGGCTCAAGGAATTGGCGTCCCCAAACGGCGCTACCAATATGGAGAGATTGGCCGCGCATTTCACTGACGCCTTGCAGGCGTTGCTCGACGGCGCTGCTTCCGGTACGTTACACG

1. *StGRAS*-10

CGCGGATCCGAACACTGCGTTTGCTGGCTTTGATG**X**TTCGGCTCAAGGAATTGGCGTCCCCAAACGGCGCTACCAATATGGAGAGATTGGCCGCGCATTTCACTGACGCCTTGCAGGCGTTGCTCGACGGCGCTGCTTCCGGTACGTTACACG

1. ***StGAMYB***
2. *StGAMYB*-1

CGCGGATCCGAACACTGCGTTTGCTGGCTTTGATGAAA**X**CACTCCAAAACCAGACAGAAAACTGGGGCTCACCTCGTTTGGCTCTTCCTTCATTAGACTCGGTTGATATTCTGATTCAGTCCCCTCCAGCTGGACATAGTGAATCCGGTAGTCTGTCACCTAGCAACA

1. *StGAMYB*-2

GTTGCTAGGTGACAGACTACCGGATTCACTGTGTCCAGCTGGAGGGGACTGAATCAGAATATCAACCGAGTCTAATGAAGGAAGAGCCAAACGAGGTGAGCCCCAGTTTTCTGTCTGGTTTTGGAGTG**X**TTTCATCAAAGCCAGCAAACGCAGTGTTCGGATCCGCG

1. *StGAMYB*-3

CGCGGATCCGAACACTGCGTTTGCTGGCTTTGATGAAA**X**CACTCCAAAACCAGACAGAAAACTGGGGCTCACCTCGTTTGGCTCTTCCTTCATTAGACTCGGTTGATATTCTGATTCAGTCCCCTCCAGCTGGACATAGTGAATCCGGTAGTCTGTCACCTAGCAACA

1. *StGAMYB*-4

CGCGGATCCGAACACTGCGTTTGCTGGCTTTGATGAAA**X**CACTCCAAAACCAGACAGAAAACTGGGGCTCACCTCGTTTGGCTCTTCCTTCATTAGACTCGGTTGATATTCTGATTCAGTCCCCTCCAGCTGGACATAGTGAATCCGGTAGTCTGTCACCTAGCAACA

1. *StGAMYB*-5

CGCGGATCCGAACACTGCGTTTGCTGGCTTTGATGAAA**X**CACTCCAAAACCAGACAGAAAACTGGGGCTCACCTCGTTTGGCTCTTCCTTCATTAGACTCGGTTGATATTCTGATTCAGTCCCCTCCAGCTGGACATAGTGAATCCGGTAGTCTGTCACCTAGCAACA

1. *StGAMYB*-6

TGTTGCTAGGTGACAGACTACCGGATTCACTATGTCCAGCTGGAGGGGACTGAATCAGAATATCAACCGAGTCTAATGAAGGAAGAGCCAAATGAGGTGAGCCCCAGTTTTCTGTCTGGTCTTGGAGTG**X**TTTCATCAAAGCCAGCAAACGCAGTGTTCGGATCCGCG

1. *StGAMYB*-7

TGTTGCTAGGTGACAGACTACCGGATTCACTATGTCCAGCTGGAGGGGACTGAATCAGAATATCAACCGAGTCTAATGAAGGAAGAGCCAAACGAGGTGAGCCCCAGTTTTCTGTCTGGTTTTGGAGTG**X**TTTCATCAAAGCCAGCAAACGCAGTGTTCGGATCCGCG

1. *StGAMYB*-8

TGTTGCTAGGTGACAGACTACCGGATTCACTATGTCCAGCTGGAGGGGACTGAATCAGAATATCAACCGAGTCTAATGAAGGAAGAGCCAAACGAGGTGAGCCCCAGTTTTCTGTCTGGTTTTGGAGTG**X**TTTCATCAAAGCCAGCAAACGCAGTGTTCGGATCCGCG

1. *StGAMYB*-9

TGTTGCTAGGTGACAGACTACCGGATTCACTATGTCCAGCTGGAGGGGACTGAATCAGAATATCAACCGAGTCTAATGAAGGAAGAGCCAAACGAGGTGAGCCCCAGTTTTCTGTCTGGTTTTGGAGTG**X**TTTCATCAAAGCCAGCAAACGCAGTGTTCGGATCCGCG

1. *StGAMYB*-10

CGCGGATCCGAACACTGCGTTTGCTGGCTTTGATGAAA**X**CACTCCAAAACCAGACAGAAAACTGGGGCTCACCTCGTTTGGCTCTTCCTTCATTAGACTCGGTTGATATTCTGATTCAGTCCCCTCCAGCTGGACATAGTGAATCCGGTAGTCTGTCACCTAGCAACA

1. *StGAMYB*-11

TGTTGCTAGGTGACAGACTACCGGATTCACTATGTCCAGCTGGAGGGGACTGAATCAGAATATCAACCGAGTCTAATGAAGGAAGAGCCAAACGAGGTGAGCCCCAGTTTTCTGTTTGGTTTTGGAGTG**X**TTTCATCAAAGCCAGCAAACGCAGTGTTCGGATCCGCG

1. *StGAMYB*-12

TGTTGCTAGGTGACAGACTACCGGATTCACTATGTCCAGCTGGAGGGGACTGAATCAGAATATCAACCGAGTCTAATGAAGGAAGAGCCAAACGAGGTGAGCCCCAGTTTTCTGTCTGGTTTTGGAGTG**X**TTTCATCAAAGCCAGCAAACGCAGTGTTCGGATCCGCG

1. ***StTm2***
2. *StTm2*-1

GTCCAACTTCCTTGGCTATGCCGAGTAAGATTTCTCCCGCTCTTGGCTGTTGTGAAACGTAGACCAGTCCAGAACACTCAAATTGATCACGAACGAGCCTATAAAGTTTCTTGGCAAG**X**TTTCATCAAAGCCAGCAAACGCAGTGTTC

1. *StTm2*-2

GTCCAACTTCCTTGGCTATGCCGAGTAAGATTTCTCCCGCTCTTGGCTGTTGTGAAACGTAGACC**X**TTTCATCAAAGCCAGCAAACGCAGTGTTC

1. *StTm2*-3

GAACACTGCGTTTGCTGGCTTTGATGAAA**X**CTACGTTTCACAACAGCCAAGAGCGGGAGAAATCTTACTCGGCATAGCCAAGGAAGTTGGAC

1. *StTm2*-4

GAACACTGCGTTTGCTGGCTTTGATGAAA**X**GGTCTACGTTTCACAACAGCCAAGAGCGGGAGAAATCTTACTCGGCATAGCCAAGGAAGTTGGAC

1. *StTm2*-5

GAACACTGCGTTTGCTGGCTTTGATGAAA**X**GGTCTACGTTTCACAACAGCCAAGAGCGGGAGAAATCTTACTCGGCATAGCCAAGGAAGTTGGAC

1. *StTm2*-6

GTCCAACTTCCTTGGCTATGCCGAGTAAGATTTCTCCCGCTCTTGGCTGTTGTGAAACGTAG**X**TTCATCAAAGCCAGCAAACGCAGTGTTCGGATCCGCG

1. *StTm2*-7

GTCCAACTTCCTTGGCTATGCCGAGTAAGATTTCTCCCGCTCTTGGCTGTTGTGAAACGTAG**X**TTCATCAAAGCCAGCAAACGCAGTGTTCGGATCCGCG

1. *StTm2*-9

GTCCAACTTCCTTGGCTATGCCGAGTAAGACTTCTCCCGCTCTTGGCTGTTGTGAAACGTAG**X**TTCATCAAAGCCAGCAAACGCAGTGTTCGGATCCGCG

1. *StTm2*-10

GTCCAACTTCCTTGGCTATGCCGAGTAAGATTTCTCCCGCTCTTGGCTGTTGTGAAACGTAG**X**TTCATCAAAGCCAGCAAACGCAGTGTTCGGATCCGCG

1. *StTm2*-11

CGCGGATCCGAACACTGCGTTTGCTGGCTTTGATGAA**X**CTACGTTTCACAACAGCCAAGAGCGGGAGAAGTCTTACTTGGCATAGCCAAGGAAGTTGGAC

1. *StTm2*-12

GTCCAACTTCCTTGGCTATGCCGAGTAAGACTTCTCCCGCTCTTGGCTGTTGTGAAACGTAG**X**TTCATCAAAGCCAGCAAACGCAGTGTTCGGATCCGCG

**Additional file: Fig. S6.** Sequencing results for 5' RLM-RACE cloning of *StARF10*, *StGRAS*, *StGAMYB* and *StTm2* in pGEMT vector are given. RACE 5' adaptor inner primer sequence is highlighted in grey in each sequence, which is followed by the target sequence. ‘X’ in target gene represents miRNA or siRNA cleavage site.
